# Supplementary material for: Glucosinolate structural diversity shapes recruitment of a metabolic network of leaf-associated bacteria
Source: Nat Commun. 2024 Oct 1;15:8496. doi: 10.1038/s41467-024-52679-7 (PMC11445407; doi:10.1038/s41467-024-52679-7)
Supplement: Supplementary file 1 — Supplementary Information [file 41467_2024_52679_MOESM1_ESM.pdf]

Supplementary Materials for:

Glucosinolate structural diversity shapes recruitment of a metabolic network of leaf-associated bacteria

Kerstin Unger[1], Ali K. Raza[1], Teresa Mayer[1#], Michael Reichelt[2], Johannes Stuttmann[3], Annika Hielscher[4], Ute Wittstock[4], Jonathan Gershenzon[2], and Matthew T. Agler[1*]

[1] Institute for Microbiology, Plant Microbiosis Group, Friedrich Schiller University Jena, Jena, Germany.
[2] Department of Biochemistry, Max-Planck Institute for Chemical Ecology, Jena, Germany.
[3] CEA, CNRS, BIAM, UMR7265, LEMiRE (Rhizosphère et Interactions sol-plante-microbiote), Aix Marseille University, 13115, Saint-Paul lez Durance, France.
[4] Institute of Pharmaceutical Biology, Technische Universität Braunschweig, Braunschweig, Germany.
[#] present address: Schülerforschungszentrum Berchtesgaden, Didactics of Life Science, Technical University of Munich, Munich, Germany.

***Corresponding author:**
Matthew T. Agler
Institute of Microbiology, Plant Microbiosis Group
Friedrich Schiller University Jena
Neugasse 23
07743 Jena, Germany
E-mail: matthew.agler@uni-jena.de
Tel: +49 (0)3641 9 49980

This PDF file includes:

Supplementary Methods
Supplementary Figures 1 to 14
Supplementary Tables 1 to 7

Supplementary Methods

Bacterial isolation, identification and growth conditions

Details on our microbial collection used in this study are in Data S1. All bacterial isolates used were recovered from *A. thaliana* leaves, the majority from leaves from our local Jena populations. For this, leaves from PB or NG2 population were sampled in spring 2018 or 2019, washed, ground and suspended in PBS + 0.02% Silwet L-77 (= leaf extract) to recover microbial leaf colonizers. In both years, we prepared near-extinction dilutions (0 to a few cells per 5 µL) of this leaf extract. In 2018, these dilutions were directly plated on R2A agar or inoculated into R2A broth to isolate bacterial strains. In 2019, the dilutions were inoculated onto axenic germinating Col-0, NG2 and PB seedlings grown on ½ MS + 0.2% sucrose + 1% agar in 24-well plates and two weeks later plants were harvested, crushed and plated to recover only efficient plant colonizers. All bacterial isolates were identified by DNA extraction using an SDS-buffer protocol: 600 µL extraction buffer (0.5 % SDS, 50 mM Tris-HCl pH=8.0, 200 mM NaCl, 2 mM EDTA in NFW) was used to resuspend a cell pellet of an overnight bacterial culture. Samples were bead beaded for 30 s at 1,500 rpm followed by 10 min incubation at 37°C. After centrifugation (20,000 x g, 5 min) the supernatant was recovered. 1/3 vol. 5M potassium acetate were added and centrifuged again, supernatants were cleaned up using 1.5x home-made magnetic Sera Mag purification beads (hereafter "magnetic beads") pre-activated and stored in PEG/NaCl as described in (*1*). PCR amplification of the 16S rRNA genes with 799F/1391R primers (2018 isolates) or 8F/1492R primers (2019 isolates) and Sanger sequencing by Eurofins Genomics (Ebersberg, Germany) was performed (all primers are listed in Supplementary Tab. 4). All isolates were cultured in R2A broth at 30°C, 150 rpm, if not stated otherwise.

Whole genome sequencing of bacterial strains of our collection of leaf colonizers

Bacterial DNA was extracted using the same SDS buffer protocol as described above. The samples were treated with RNAse A (2 µg/mL, 37°C for 30 min; ≥ 80 U/mg (Kunitz), ITW Reagents, Italy), followed by a treatment with Proteinase K (100 µg/mL, 37°C for 1 h; Proteinase K from *Tritirachium album*, ≥ 30 U/mg, Sigma Aldrich, Germany). We then purified the samples twice by adding an equal volume of phenol/chloroform/isoamyl alcohol and recovering the aqueous layer. The DNA was precipitated by adding sodium acetate and ethanol overnight. In total we sent 30-40 µL with a DNA concentration of at least 50 ng/µL for whole genomes sequencing. The

genomes were sequenced on a NextSeq 2000 platform (Illumina Sequencing) at a depth of 300 MBp at Microbial Genome Sequencing Center (Pittsburgh, PA 15222, USA). The results were provided as paired end reads (2x151bp, fastq files). We assembled and annotated the sequences using an in-house perl script. We trimmed the raw reads with the software TrimmomaticPE (*2*) and then assembled them with the software SPAdes Version 3.14.1 (*3*) using default k-mer sizes of 21, 33, 55 and 77. The resulting scaffolds fasta file was then annotated with PROKKA 1.14.6 (*4*) and the reference genome Pseudomonas_syringae_pv_tomato_DC3000_111.gbk retrieved from NCBI database.

<u>Homology search of bacterial myrosinases and ITC hydrolases in whole genome of strain R3</u>

Genomic DNA was extracted from overnight cultures of R3 in R2A broth using the QIAGEN MagAttract HMW DNA Kit for gram-negative bacteria according to the manufacturer's instructions. The gDNA was sent to Eurofins Genomics (Koblenz, Germany) for Oxford Nanopore sequencing. The genome was assembled by Eurofins into three contigs, the assembly was 100% complete with 0.04% contamination. Based on sequence homology strain R3 was identified as *Serratia* sp. (98.37% sequence identity). In total 4697 coding sequences were identified. To search for homologs to bacterial myrosinases and bacterial ITC hydrolases we generated two databases with known protein sequences. We selected all bacterial ITC hydrolases (SaxA) which were identified and functionally characterized by (*5*) and collected amino acid sequences of bacterial myrosinases from plant and soil environments which were shown to be functionally active to generate a myrosinase database (see Supplementary Data 3). We performed a BLAST homology search (e-value < 0.001) based on all protein sequences from R3 against both databases. Next, we checked for secretion signals using SignalP 6.0 (*6*).

<u>Analysis of 4MSOB-ITC and 4MSOB-amine in bacterial cultures by LC-MS/MS</u>

4MSOB-ITC in bacterial cultures (n = 3) was analyzed on an Agilent 1200 HPLC system (Agilent, Santa Clara, CA, United States) coupled to an API3200 tandem mass spectrometer (AB SCIEX, Darmstadt, Germany). Non-inoculated medium served as control (n = 3). Amines were separated by an Agilent XDB-C18 column (5 cm × 4.6 mm, 1.8 µm, Agilent, Waldbronn, Germany). The mobile phase consisted of 0.05% (v/v) formic acid in ultrapure water as solvent A and acetonitrile as solvent B, at a flow rate of 1.1 mL/min. The elution gradient was: 0-0.5 min, 3-15% B; 0.5-2.5

min, 15-85% B; 2.5-2.52 min, 85-100% B; 2.25-3.5 min, 100% B; 3.5-3.51 min, 100-3% B, 3.51-6 min, 3% B. The ion spray voltage was maintained at 5500 eV in positive mode. The turbo gas temperature was set to 500 °C, nebulizing gas to 60 psi, drying gas to 60 psi, curtain gas to 35 psi, and collision gas to 3 psi. Details of multiple reaction monitoring (MRM) can be found in Supplementary Tab. 7. Analyst Software 1.6 Build 3773 (AB SCIEX) was used to acquire and process data.

Detailed analysis of GLSs of leaves and bacterial cultures

For leaf material, the measurement was performed as previously described by (*7*). After freeze-drying, three to five rosettes per genotype were ground to powder by shaking with metal beads. GLSs were extracted from 8-12 mg of the powder by 1 mL of 80 % MeOH with 50 µM pOH-Benzyl GLS (Sinalbin, internal standard) and thorough vortexing for 30 sec and shaking for 30 min at high speed. The samples were centrifuged for 5 min at 3,500 x g and 600 µL or 800 µL of the supernatant was added to a freshly prepared DEAE-Sephadex-filter plate and allowed to flow through. Samples on the DEAE-Sephadex were washed five times with: (1) 0.5 mL 80% MeOH, (2/3) two times 1 mL ultrapure water, (4) 0.5 mL 0.02 MES pH 5.2 and (5) 30 µL sulfatase solution. Samples were kept at room temperature overnight and desulfo- (ds)-GLSs were eluted with 0.5 mL ddwater on the next day. The eluted ds-GLSs were separated using high performance liquid chromatography (Agilent 1100 HPLC system, Agilent Technologies) on a reversed phase column (Nucleodur Sphinx RP, 250 x 4.6 mm, 5µm, Macherey-Nagel, Düren, Germany) with a water (A)-acetonitrile (B) gradient (0-1 min, 1.5% B; 1-6 min, 1.5-5% B; 6-8 min, 5-7% B; 8-18 min, 7-21% B; 18-23 min, 21-29% B; 23-23.1 min, 29-100% B; 23.1-24min 100% B and 24.1-28 min 1.5% B; flow 1.0 mL min-1). Detection was performed with a photodiode array detector and peaks were integrated at 229 nm. We used the following response factors: 3OHP and 4OHB 2.8, all other aliphatic GLS 2.0, indole GLS 0.5 (*8*) for quantification of individual GLSs. The identity of the peaks was based on a comparison of retention time and UV absorption spectrum with data obtained for isolated ds-GLSs as described in (*9*) and by analysis of the ds-GLS extracts on an LC-ESI-Ion-Trap-mass spectrometer (Esquire6000, Bruker Daltonics).

To quantify GLSs on the leaf surface a leaf dipping assay similar to (*10*) was performed. Per sample one leaf of NG2, NG*myb28*, Col-0 or *myb28/29* plants was submerged in methanol and gently swirled for 30 s in the solvent. An obvious rupture of plant cells was detectable when the

methanol turned green, these samples were excluded from the experiment. In the end four leaves per genotype were sampled. All methanol was collected and the levels of GLSs in the leaf dip samples were quantified after spiking the extract with the internal standard sinigrin (Carl Roth, Mannheim, Germany; concentration 5 µM) as described in the next paragraph for bacterial cultures. Compounds were quantified using the internal standard and applying a theoretical response factor of 1 for all GLSs. Analyst Software 1.6 Build 3773 (AB SCIEX) was used to acquire and process data.

For bacterial culture supernatants, after centrifugation at max. speed the supernatants were recovered from three replicate cultures per treatment, non-inoculated medium served as control. The levels of GLSs were quantified in 1:10 (v:v) diluted extracts using an Agilent 1200 HPLC system (Agilent, Santa Clara, CA, United States) coupled to an API3200 tandem mass spectrometer (AB SCIEX, Darmstadt, Germany) according to (*11*). GLSs were separated on an EC 250/4.6 NUCLEODUR Sphinx RP column (250 mm × 4.6 mm, 5 µm; Macherey-Nagel, Düren, Germany). The mobile phase consisted of 0.2% (v/v) formic acid in ultrapure water as solvent A and acetonitrile as solvent B, at a flow rate of 1 mL/min. The elution gradient was: 0-1 min, 1.5% B; 1-6 min, 1.5-5% B; 6-8 min, 5-7% B; 8-18 min, 7-21% B; 18-23 min, 21-29% B; 23-23.1 min, 29-100% B; 23.1-24 min, 100% B; 24-24.1 min, 100-1.5% B; 24.1-28 min, 1.5% B. The ionization source was set to negative mode. The ion spray voltage was maintained at -4,500 eV. Gas temperature was set to 500 °C, nebulizing gas to 60 psi, drying gas to 60 psi, curtain gas to 30 psi and collision gas to 6 psi. For each compound, the transitions from precursor ion to product ion was monitored using multiple reaction monitoring (MRM) (Supplementary Tab. 6). Compounds were quantified using external calibration curves generated using the following compounds: Allyl-GLS (Carl Roth, Mannheim, Germany), 4-methylsulfinylbutyl-GLS (4MSOB-GLS, Phytoplan, Heidelberg, Germany), and 2OH3But-GLS (Progoitrin, Phytoplan, Heidelberg, Germany). Analyst Software 1.6 Build 3773 (AB SCIEX) was used to acquire and process data.

Detailed analysis of GLS breakdown products in leaf homogenates and bacterial cultures

For leaf homogenates three rosettes were sampled per genotype. Internal standards (25 µL of 100 ng/µL phenyl cyanide in methanol, 20 µL of 1 nmol/µL indole-3-carbonitrile in methanol) were added to each sample after crushing the leaf material in MES buffer. After 10 min total incubation time, the samples were centrifuged at 20,000 x g at 20°C for 10 min. The supernatant was

transferred into 1.5 mL glass vials and frozen at -20°C until further processing. Thawed supernatants were extracted twice with 750 µL dichloromethane. The organic phases were pooled and dried over Na_2SO_4, concentrated in an air stream to about 150 µL and analyzed by GC-MS and GC-FID for aliphatic hydrolysis products as described in (*12*), using a ZB-5MS capillary column (30 m × 0.25 mm; ft 0.25 µm; Phenomenex, Aschaffenburg, Germany).

To analyze GLS breakdown product generated by R3, 200 µL cultures in M9 medium with 10 mM allyl-GLS were grown for 150 h and with 10 mM 2OH3But-GLS for 220 h in a 96-well plate as described above. Non-inoculated medium served as control (n=3). Each condition consisted of three replicate wells. 10 µL of the supernatants were diluted 1:10 in ddwater to measure amines after pre-tests had shown too high peaks of undiluted samples. Amines were analyzed on an Agilent 1200 HPLC system (Agilent, Santa Clara, CA, United States) coupled to an API3200 tandem mass spectrometer (AB SCIEX, Darmstadt, Germany). Amines were separated by an Agilent XDB-C18 column (5 cm × 4.6 mm, 1.8 µm, Agilent, Waldbronn, Germany). The mobile phase consisted of 0.05% (v/v) formic acid in ultrapure water as solvent A and acetonitrile as solvent B, at a flow rate of 1.1 mL/min. The elution gradient was: 0-0.5 min, 3-15% B; 0.5-2.5 min, 15-85% B; 2.5-2.52 min, 85-100% B; 2.25-3.5 min, 100% B; 3.5-3.51 min, 100-3% B, 3.51-6 min, 3% B. The ion spray voltage was maintained at 5500 eV in positive mode. The turbo gas temperature was set to 500 °C, nebulizing gas to 60 psi, drying gas to 60 psi, curtain gas to 35 psi, and collision gas to 3 psi. For each compound, the transitions from precursor ion to product ion was monitored using multiple reaction monitoring (MRM) (Supplementary Tab. 7). Compounds were quantified using an external calibration curve generated with allyl-amine (Sigma-Aldrich, Taufkirchen, Germany). Analyst Software 1.6 Build 3773 (AB SCIEX) was used to acquire and process data.

10 µL of phenyl cyanide (1:10000 dilution in MeOH; Merck, Darmstadt, Germany) was added as internal standard to 140 µL of the supernatant. Nonpolar compounds were extracted with 400 µL dichloromethane, vortexing and centrifugation for 1 min at 3,500 x g. About 100 µL of the organic phase was transferred into fresh glass vials. Allyl-ITC and goitrin were quantified following the method described in (*13*). Samples were analyzed by GC-FID using an Agilent 6890 Series gas chromatograph with an DB5MS column (30 m x 0.25 mm x 0.25 m film, Agilent Technologies), splitless injection at 200°C, and a temperature program of 35°C for 3 min, a 12°C/min ramp to 96°C and a 18°C/min ramp to 240°C (with a 6 min final hold). Peaks were identified by

comparison of retention times and mass spectra to those of the authentic standards, allyl-ITC (Fluka), and goitrin (Sigma-Aldrich, Taufkirchen, Germany). Hydrogen was used as the carrier gas and the detector operated at 300°C. Quantification was based on peak area relative to that of the internal standard, phenyl cyanide. Response factors (RF) relative to phenyl cyanide were experimentally determined for allyl-ITC (1.55) and calculated for goitrin (1.79) by the effective carbon number (ECN) concept (*14*).

Detailed non-target metabolite analysis by LC-ESI-Q-ToF-MS

For non-target analysis of R3 mono-culture and co-cultures of R3 with Ps9 and/or J4, ultra-high-performance liquid chromatography–electrospray ionization– high resolution mass spectrometry (UHPLC–ESI–HRMS) was performed with a Dionex Ultimate 3000 series UHPLC (Thermo Scientific) and a Bruker timsToF mass spectrometer (Bruker Daltonik, Bremen, Germany) as described in (*15*). Each treatment consisted of three replicate wells, however, R3 mono-culture has not grown in one replicate and we excluded it from further analysis. Non-inoculated medium served as control (n = 3). UHPLC was used applying a reversed-phase Zorbax Eclipse XDB-C18 column (100 mm × 2.1 mm, 1.8 µm, Agilent Technologies, Waldbronn, Germany) with a solvent system of 0.1% formic acid (A) and acetonitrile (B) at a flow rate of 0.3 ml/min. The elution profile was the following: 0 to 0.5 min, 5% B; 0.5 to 11.0 min, 5% to 60% B in A; 11.0 to 11.1 min, 60% to100% B, 11.1 to 12.0 min, 100% B and 12.1 to 15.0 min 5% B. HRMS analyses were performed separately with positive and negative ionization and automatic MS2 scans ("autoMS", data-dependent acquisition) enabled. The mass spectrometer parameters were set as follows: capillary voltage 4.5 KV/-3.5KV, end plate offset of 500V, nebulizer pressure 2.8 bar, nitrogen at 280°C at a flow rate of 8L/min as drying gas. Acquisition was achieved at 12 Hz with a mass range from m/z 50 to 1500, with data-dependent MS/MS and an active exclusion window of 0.1 min, a reconsideration threshold of 1.8-fold change, and an exclusion after 5 spectra. Fragmentation was triggered on an absolute threshold of 50 counts and acquired on the two most intense peaks with MS/MS spectra acquisition of 12 Hz. Collision energy was alternated between 20 and 50V. At the beginning of each chromatographic analysis 10 µL of a sodium formate-isopropanol solution (10 mM solution of NaOH in 50/50 (v/v%) isopropanol water containing 0.2% formic acid) was injected into the dead volume of the sample injection for re-calibration of the mass spectrometer using the expected cluster ion m/z values.

Peak detection was carried out using Metaboscape software (Bruker Daltonik, Bremen, Germany) with the T-Rex 3D algorithm for qTOF data. For peak detection the following parameters were used: intensity threshold of 1000 with a minimum of 10 spectra, time window from 0.4 to 12 min, peaks were kept if they were detected in at least 60% of all replicates of one sample group. Adducts of [M+H]+, [M+Na]+, [M+K]+, and [2M+H]+ in positive mode, and [M-H]-, [M+Cl]-, [M+COOH]-, and [2M-H]- in negative mode were grouped as a single bucket if they had an EIC correlation of 0.8. For feature identification the MS-MS spectra data were uploaded both to GNPS using the feature based molecular network (FBMN) workflow, on the online platform (*16*) (https://gnps.ucsd.edu) and SIRIUS software v5.8.6 (*17*) (https://bio.informatik.uni-jena.de/sirius/). SIRIUS predicted putative formulas for each feature, which were ranked using the ZODIAC algorithm (*18*). The CANOPUS algorithm of SIRIUS was additionally applied for compound class predictions (*19*). Additionally, we compared MS/MS spectra to an in-house database to confirm or identify further compounds. For statistical analysis we focused on comparing the monoculture R3 to co-cultures and performed pairwise t-tests for these comparisons to identify significantly different features in R (R version 4.3.0). Only features with $p<0.05$ in at least one of these comparisons and only those features which were not artefacts of allyl-GLS were selected. If a compound showed up in positive and negative mode, we selected to show the results of the one with the higher peak. The significant features were log10 transformed and plotted in comparison to the non-inoculated control and a Venn diagram was generated to show communalities and differences between the communities.

Feature tables in positive and negative mode, p-values of pairwise comparisons along with our analysis pipeline using R and annotations of significant features are provided in the manuscript's figshare folder (see Data Availability section).

DNA extraction from enrichment cultures (dataset 1 and 4)

Bacterial DNA from GLS enrichments (dataset 1, Supplementary Tab. 5) was extracted from glycerol stocks (dataset 1) or frozen cell pellets of the cultures (dataset 4) using the same SDS buffer protocol as described earlier for individual bacterial strains. To recover higher quality DNA the resulting supernatants were cleaned up using RNAse A (10 µg/mL, 30 min at 37°C) and proteinase K treatment (100 µg/mL, 1 h at 37°C) (left out for dataset 4), followed by a two-step purification: equal amounts of phenol/chloroform were added to the supernatants, followed by

centrifugation at 20,000 x g for 10 min at 4°C and recovery of the aqueous phase. Next, equal amounts of chloroform/isoamyl alcohol (24:1) were added, followed by centrifugation at 20,000 x g for 10 min at 4°C and recovery of aqueous phase. The DNA was precipitated by adding 1/10 Vol. sodium acetate (3M, pH5) and 2.5 Vol. isopropanol or ethanol, incubation at -20°C overnight and 30 min centrifugation at 4°C at 20,000 x g. The DNA pellet was washed twice with 80% ethanol and air-dried before resuspending in 10 mM Tris-HCl pH8.

DNA extraction from lab and wild plants (datasets 2 and 3)

To extract DNA from plant material of wild *A. thaliana* plants for dataset 3 (Supplementary Tab. 5), all tubes with leaf material were bead beat for 30 s at 1,400 rpm. 150 µL of 2x CTAB buffer (2% cetyltrimethylammonium bromide, 1% polyvinylpyrrolidone, 100 mM Tris-HCl, 1.4 M NaCl, 20 mM EDTA) was added to each tube, vortexed briefly and incubated at 37°C for 10 min. The tubes were spun down for 5 min at 20,000 x g and the supernatant was transferred into a deep 96-well plate. 1/10 Vol. of sodium acetate (3M, pH5) and 2.5 Vol. of 100% ethanol were added to each sample and the plate was frozen at -20°C to precipitate DNA overnight. To collect the DNA the plate was centrifuged at 20,000 x g for 60 min at 4°C. All supernatants were discarded, and the pellets were washed twice with 70% ethanol, airdried and re-suspended in 50 µL 10 mM Tris-HCl (pH8). The samples were frozen at -20°C overnight and cleaned up using 1.5x magnetic beads as described earlier. To extract DNA of lab-grown plants (dataset 2), all tubes with leaf material were bead beat for 30 s at 1,400 rpm. 300 µL of 2x CTAB buffer was added to each tube, vortexed briefly and incubated at 65°C for 15 min. The tubes were spun down for 5 min at 20,000 x g and the supernatant was transferred into a fresh tube. Equal amounts of Phenol:Chloroform:Isoamyl alcohol (25:24:1) were added, briefly vortexed and centrifuged for another 5 min at 20,000 x g. The DNA was precipitated and eluted as described for data set 3, without additional magnetic bead clean-up.

Detailed amplicon sequencing procedure

Master mixes for the first PCR for dataset 2 and 3 contained 11.36 µL nuclease-free water, 4.00 µL 5x Kapa High Fidelity Buffer, 0.60 µL 10 mM Kapa dNTPs, 0.16 µL of each primer (GI_F, GI_R, 341F-OH, 799R-OH, Supplementary Tab. 4), 0.50 µL of each blocking Oligo (At_BLc_16S_F5, At_BLc_16S_R1, Supplementary Tab. 4), 0.40 µL Kapa HiFi polymerase

(Kapa Biosystems), 2.00 µL normalized DNA (50-100 ng/µL) as template per reaction. Blocking oligos bound to their targets in the first PCR in a BioRad thermocycler: (1) 95°C for 3:00 min, (2) 98°C for 0:20 min, (3) 58°C for 0:30 min, (4) 55°C for 1:00 min, (5) 72°C for 1:00 min, to step (2) and repeat for 5 cycles, (6) 72°C for 2:00. All samples were cleaned up enzymatically by adding 0.50 µL Exonuclease I (NewEngland Biolabs), 0.50 µL Antarctic Phosphatase and 1.22 µL Antarctic Phosphatase Buffer (NewEngland Biolabs) to 10 µL of the first PCR reaction at 37°C for 30 min, followed by enzyme deactivation at 80°C for 15 min. In the second PCR the master mix consisted of 7.34 µL nuclease-free water, 4.00 µL Kapa High Fidelity Buffer, 0.6 µL 10 mM dNTPs, 0.40 µL Kapa polymerase, 5 µL of cleaned first PCR reaction, along with unique forward and reverse indexing primers for each sample. Products were amplified: (1) 95°C for 3:00 min, (2) 98°C for 0:20 min, (3) 60°C for 1:00 min, (4) 72°C for 1:00 min, to step (2) and repeat for 35 cycles, (5) 72°C for 2:00. All samples were cleaned up using 1.5x vol. magnetic beads as described earlier in the methods.

To pool the libraries of datasets 2 and 3, the fluorescence of each sample was measured with Picogreen (1:200 diluted stock, Quant-iT™ PicoGreen™, ThermoFisher) in a qPCR machine (qTower^3, JenaAnalytik, Jena, Germany). Samples were pooled according to their normalized fluorescence relative to the highest fluorescent well. Dataset 2 contained plant GI reads and the entire volume of the final library ran on a 2 % high resolution agarose gel to separate GI and 16S bands. Both were purified from the gel using the GeneJET gel purification kit (ThermoFisher). Additionally, we re-barcoded the entire library using 1-5 ng of the pooled library according to (*20*) with minor modifications. The PCR master mix contained 7.34 µL nuclease-free water, 4.00 µL 5x Kapa Buffer, 0.60 µL 10 mM Kapa dNTPs, 0.40 µL Kapa enzyme, 1.33 µL of 4.5 µM of forward and reverse indexing primers and 5 µL of the final pool. Products were amplified in 8 cycles with the protocol for the second PCR mentioned above for dataset 2. Afterwards the re-barcoded library was cleaned up using 1.5x magnetic beads. In the end, the fluorescence of the two fractions (GI, 16S) along with the re-barcoded library was determined and the fractions were combined (94% 16S rRNA, 5% GI, 1% re-barcoded library).

For bacterial-only communities from GLS enrichments (dataset 1 and 4) the PCR protocols were the same as mentioned above with minor exceptions. As there is no need to block plant 16S reads or quantify GI reads we only used 341F-OH/799R-OH primers for amplification, instead the volume of DNA template was increased to 2.5 µL per 10 µL reaction (dataset 1). Because of

unspecific bands after the second PCR, pooling was performed according to the brightness of the 16S rRNA gene bands (660 bp) on a gel using ImageJ. The samples were normalized relative to the brightest sample and pooled into sub-pools. Libraries were sequenced on a Illumina MiSeq instrument for either 600 cycles (dataset 2,3) or 300 cycles (dataset 1,4).

<u>Analysis of diversity of bacterial communities based on 16S rRNA gene data</u>

Richness and evenness (Chao1, Shannon, Simpson, ACE) as alpha diversity measures were calculated in datasets 1 and 2. To plot bar charts and calculate beta diversity matrices we excluded samples with less than 100 bacterial reads in all three datasets. For the bacterial communities in the enrichments (dataset 1), we agglomerated the taxa at genus level and taxa with less than 0.02 % abundance in one sample were agglomerated and classified as “Remainder” in the bar charts. In dataset 3 it was necessary to remove Brassicales plants from non-arabidopsis samples. We used the 16S reads derived from the plant (Phylum “Streptophyta”) to identify all plants at the order level (this mostly included Brassicales, Asterales, and Poales). For “other” plants, we applied a conservative approach wherein if a majority of plant reads were assigned to Brassicales, that sample was discarded. For dataset 4 we agglomerated on family level, calculated relative abundances and depicted stacked bar charts by adding the abundances of the inoculated families (Yersiniaceae, Oxalobacteriaceae, Pseudomonadaceae). For dataset 1 we used relative abundance data to calculate beta diversity distances, and dataset 2 was normalized to plant GI reads. In both cases, beta diversity is based on the Bray-Curtis and Jaccard distance and ordination was performed with principle coordinate analysis (PCoA). For visualization purposes we constrained by genotype. The statistical significance of explaining variables was tested with a permutational analysis of variance (PERMANOVA) test. For dataset 3, we eliminated samples with fewer than three biological replicates per treatment and agglomerated on genus level. Beta diversity was calculated as the Aitchison distance between samples, which applies a centered log ratio (CLR) transformation followed by calculating Euclidean distances. The resultant patterns were illustrated in ordination plots that were faceted by year and plant type to provide a comprehensive overview. The influence of various factors, including plant type, location, year, and month, as well as the interaction of the variables on microbial community structures was assessed using PERMANOVA (significance level $p<0.05$).

To perform differential abundance analysis (DESeq2 (*21*)) on pairwise comparisons, we agglomerated on genus, family or order level. In any case we added a pseudo count of 1 to each read in the ASV table of non-normalized counts in dataset 3 or plant GI-normalized reads in dataset 2. Size factors were estimated based on the geometric means of each taxon. A Wald test with a parametric fit was executed to determine significant differences. To control the false discovery rate, the Benjamini-Hochberg method was adopted. Taxa with adjusted p-values below 0.05 were marked as significantly differentially abundant. log10-transformed abundances were plotted and a Wilcox test was applied to confirm the significance.

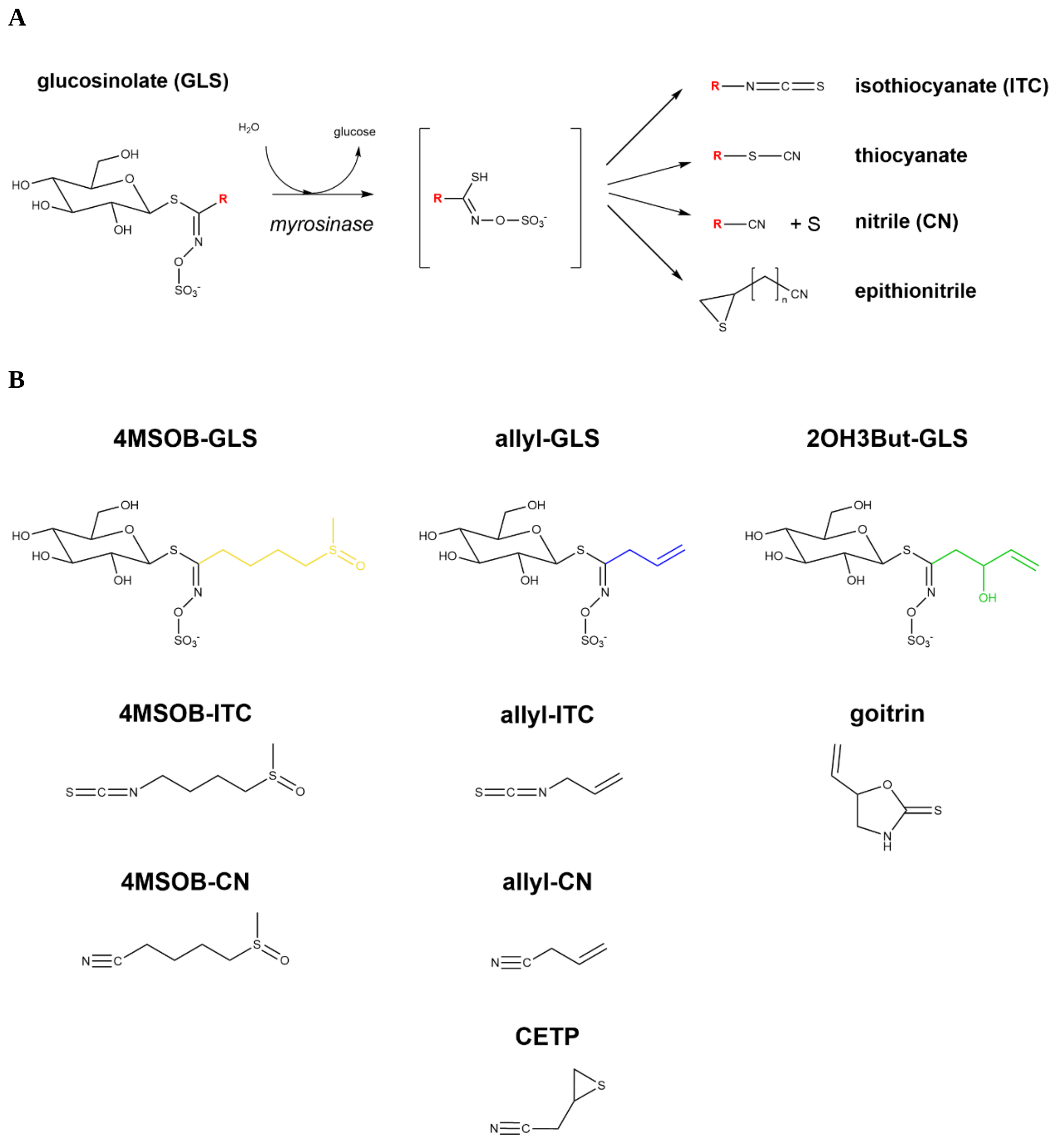

Supplementary Figure 1.

Chemical structures of GLSs and their breakdown products detected in this study. (**A**) Schematic drawing of GLS hydrolysis. A myrosinase hydrolyzes the GLSs, the unstable aglycone rearranges to different possible breakdown products. Which product is formed depends on genetic factors of the plant (nitrile-specifier protein, epithiospecifier protein) and environmental conditions (e.g. pH, temperature). (**B**) Chemical structures of 4-methylsulfinylbutyl glucosinolate (4MSOB-GLS, in Col-0 genotype), allyl-GLS (in NG2 genotype) and 2-hydroxy-3-butenyl glucosinolate (2OH3But-GLS, in Woe genotype) and their corresponding breakdown products which are relevant for this study: 4MSOB-ITC, allyl-ITC (isothiocyanates); 4MSOB-CN, allyl-CN (nitriles), CETP (3,4-epithiobutanenitrile) and goitrin.

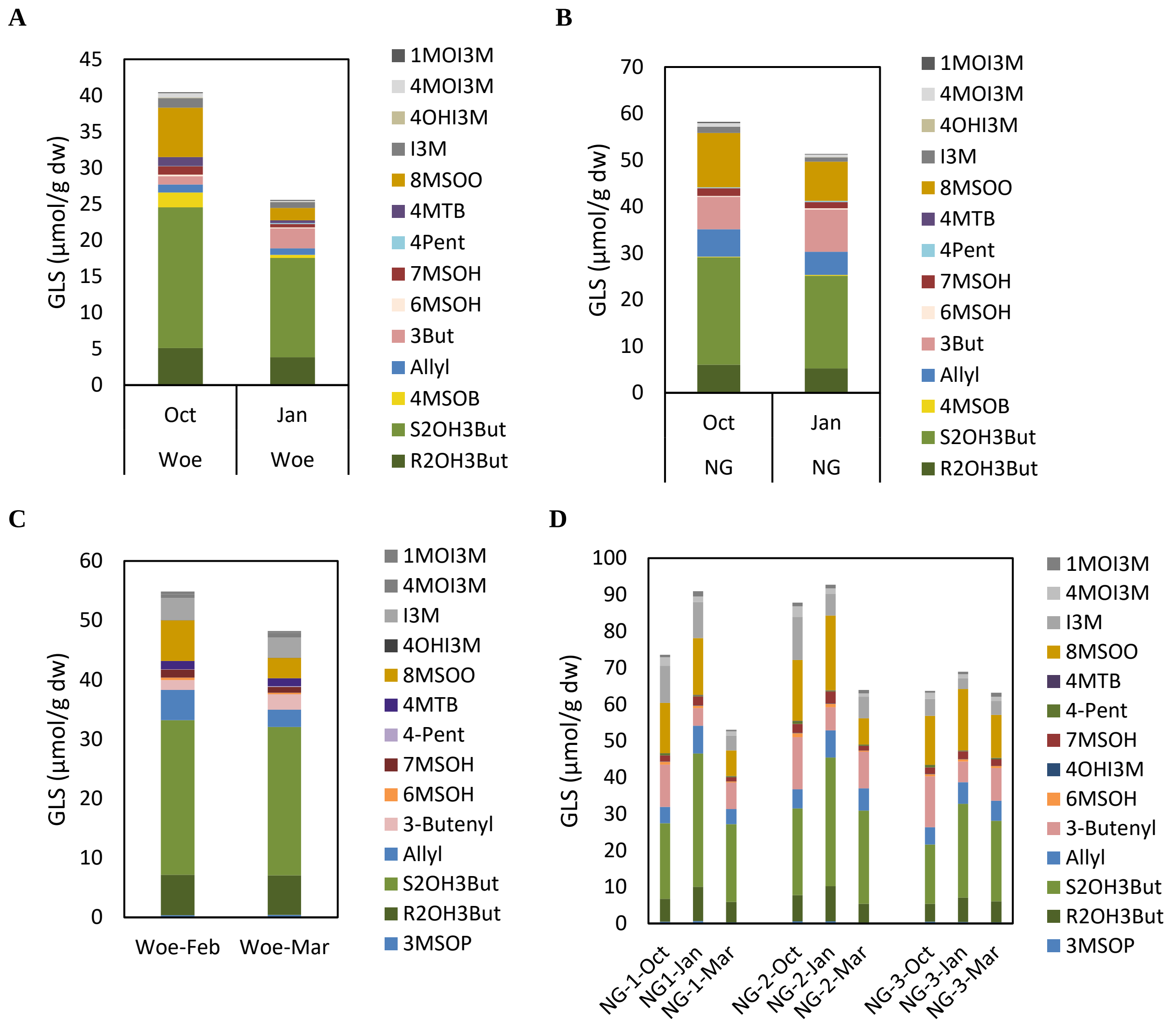

Supplementary Figure 2.

GLS profiles of wild *A. thaliana* populations NG2 and Woe. Average GLS concentration of 5-6 replicates sampled at Woe (**A**) and NG2 (**B**) in October 2021 and January 2022. (**C**) Average GLS concentration of 5-6 replicates of Woe sampled in February and March 2023. (**D**) Average GLS concentration of 5 replicates of three sub-plots at NG2 location sampled in October 2022, January and March 2023. Abbreviations of GLSs are listed in the Supplementary methods section. Underlying data is available in our figshare folder (see Data Availability).

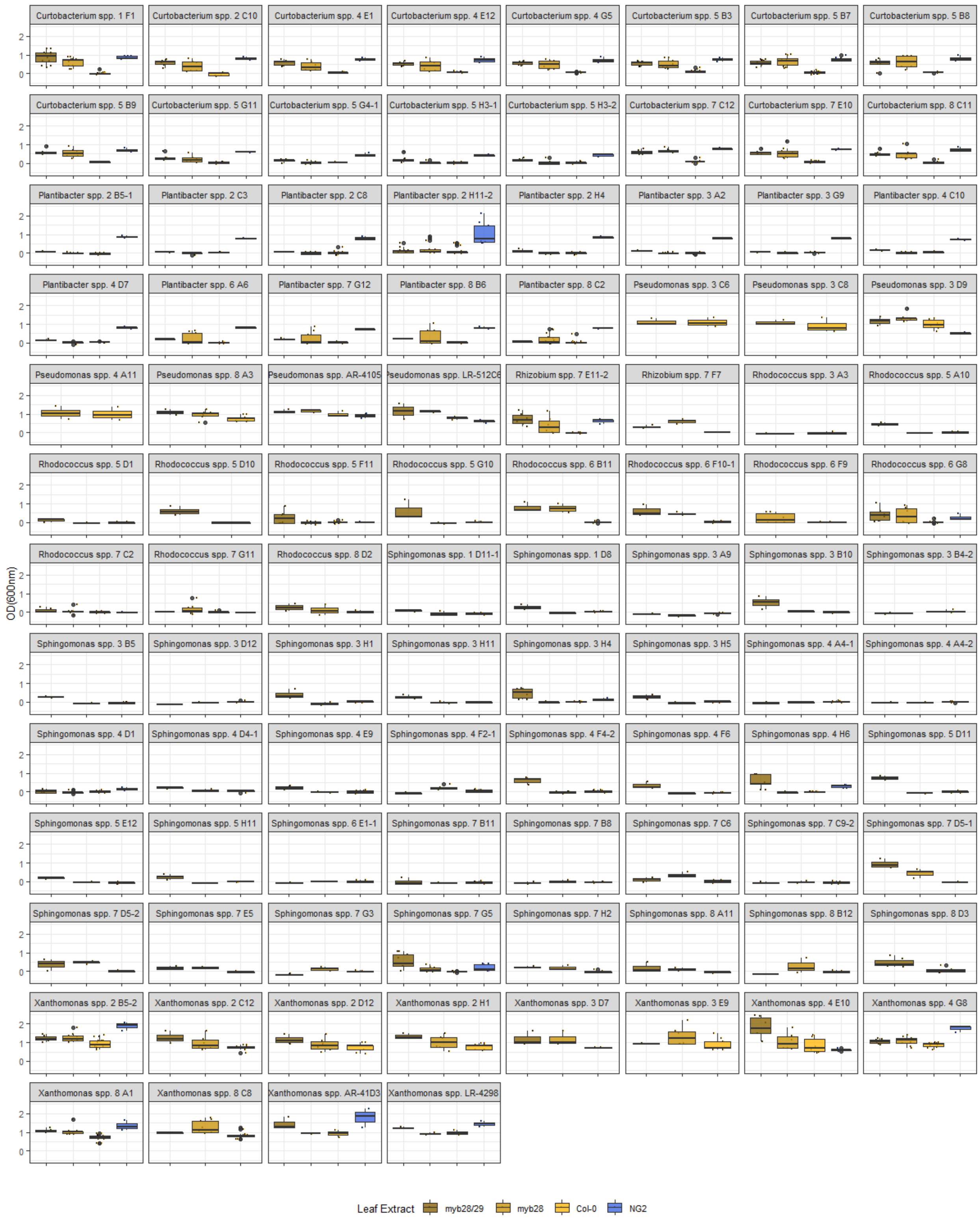

Supplementary Figure 3.

Bacterial growth in leaf extract medium after 24-96 h incubation time. The plots represent individual results for all strains which are shown on genus level in Fig. 2A. Number of samples per condition are provided in Supplementary Data 4. Additionally, all data is available in our figshare folder (see Data Availability).

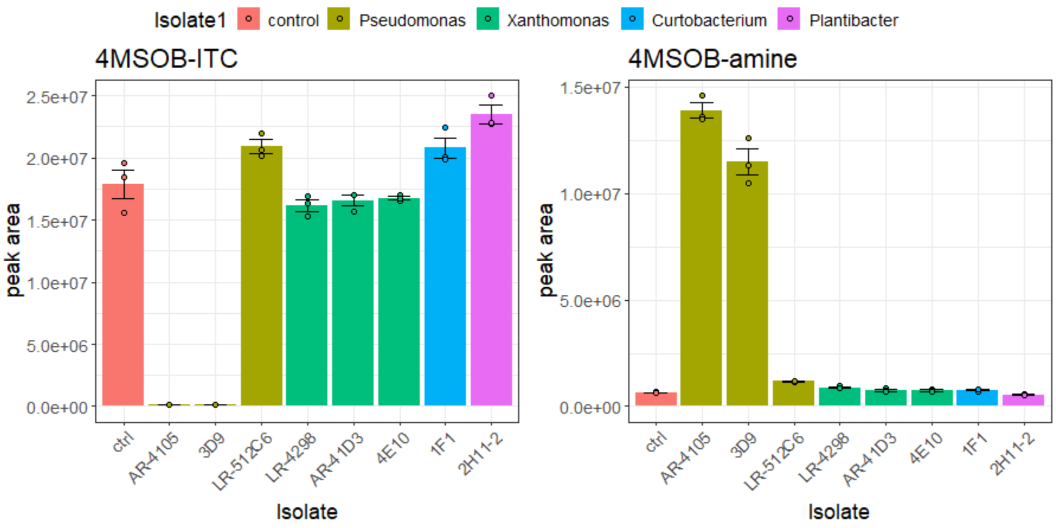

Supplementary Figure 4.

Bacterial 4MSOB-ITC degradation *in-vitro*. Bacterial strains were grown for 18 h in R2A broth supplemented with 30 µg/mL 4MSOB-ITC, non-inoculated medium served as control (n=3). 4MSOB-ITC and its *saxA*-mediated breakdown product 4MSOB-amine were measured in the aqueous supernatants without further extraction. Mean and standard error of the mean of the peak area of 4MSOB-ITC and 4MSOB-amine from LC-MS/MS of the same replicates. Overlaid dots visualize individual replicates. All data is available in our figshare folder (see Data Availability).

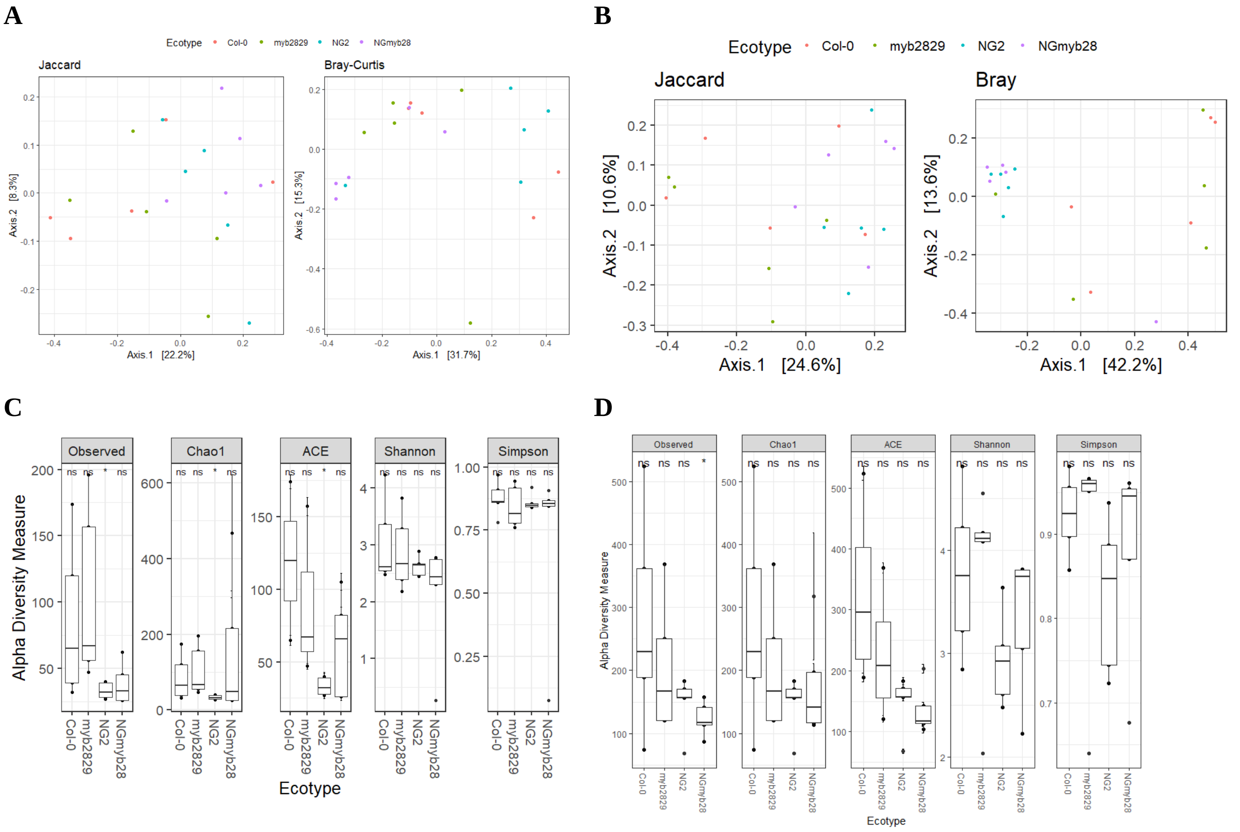

Supplementary Figure 5.

Alpha and beta diversity of leaf bacterial communities of lab-grown NG2, NG*myb28*, Col-0 and *myb28/myb29* plants. Plants were grown for 3-4 weeks and bacterial communities were assessed in either surface sterilized (**A,C**) or washed leaves (**B,D**). 5 replicate rosettes per genotype with >100 reads per sample were included in the analysis. To calculate beta diversities, the data was agglomerated on genus level, and reads were normalized to the plant GI reads. Detailed statistical analyses for all plots are presented in Supplementary Data 2. (**A**) Unconstraind PCoA plots of beta diversity (Jaccard, Bray-Curtis) in endophytic community data. (**B**) Unconstrained PCoA plots of beta diversity (Jaccard, Bray-Curtis) in total community data. (**C**) Alpha diversity of non-normalized samples for endophytic communities. (**D**) Alpha diversity of non-normalized samples for total communities. All data is available in our figshare folder (see Data Availability).

A

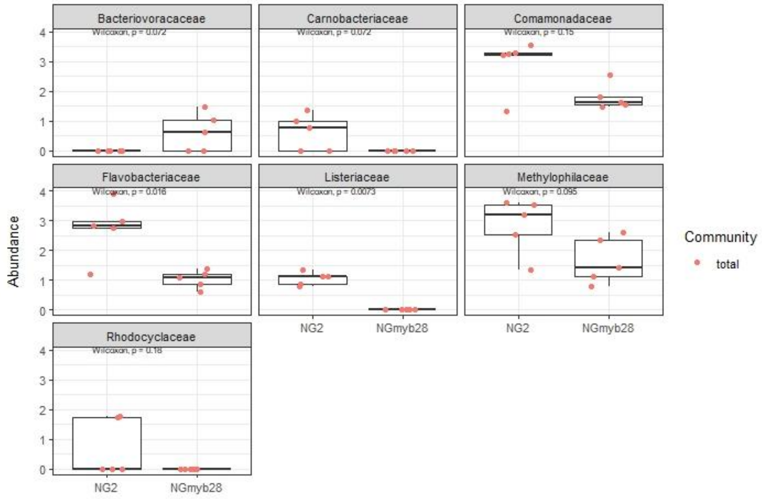

B

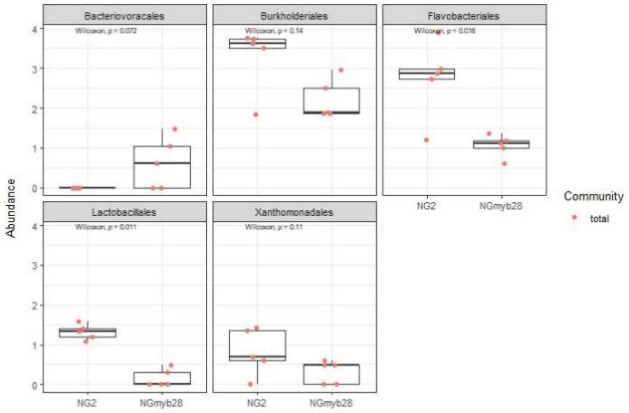

Supplementary Figure 6.

DESeq analysis of leaf bacterial communities of lab-grown NG2 and NG*myb28* plants. Differentially abundant taxa on NG2 compared to NG*myb28* leaves using DESeq analysis with a cutoff of alpha = 0.05. 5 replicate rosettes with >100 reads per sample were included in the analysis. Significant taxa were log10-transformed, and a more stringent Wilcoxon test (two-sided, no p-adjustment) was used to provide additional information on the strength of the enrichment. ASVs were agglomerated on family (**A**) and order (**B**) levels. All data is available in our figshare folder (see Data Availability).

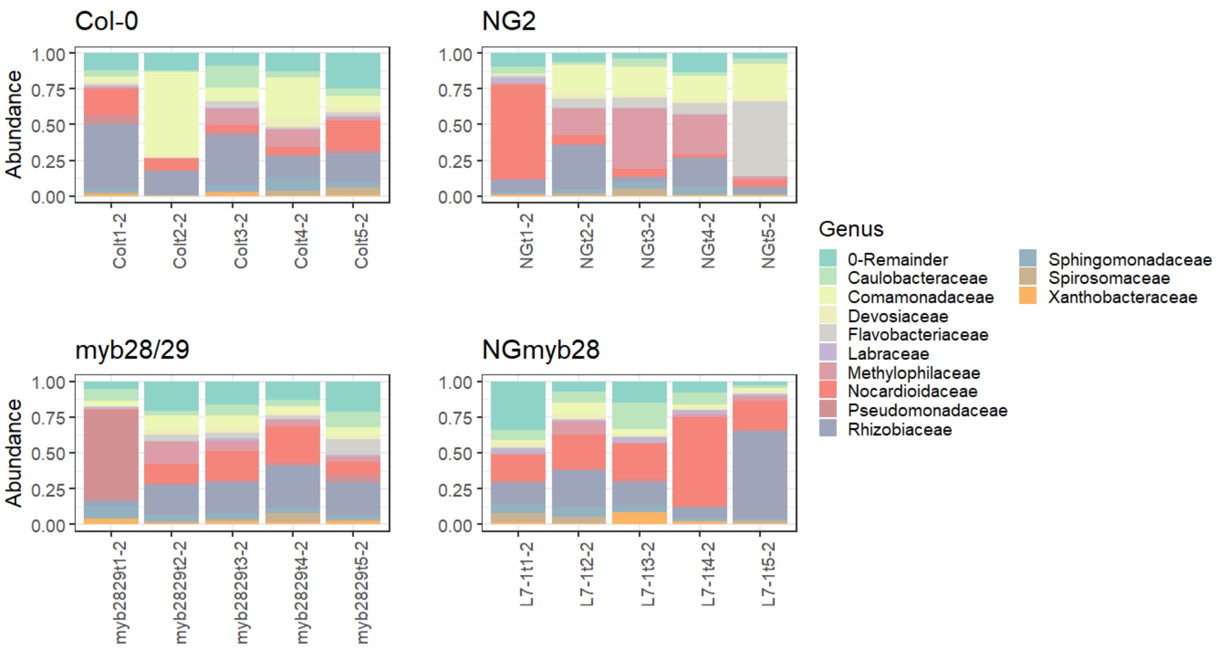

Supplementary Figure 7.

Relative abundance of total bacterial communities of lab-grown NG2, NG*myb28*, Col-0 and *myb28/29* plants. The bar charts show the community composition assessed by 16S rRNA gene amplicon sequencing. Each bar represents one replicate rosette (n=5). ASVs were agglomerated on family level and families below 0.02% abundance were merged and classified as "Remainder". Only replicates with >100 reads were considered. All data is available in our figshare folder (see Data Availability).

A

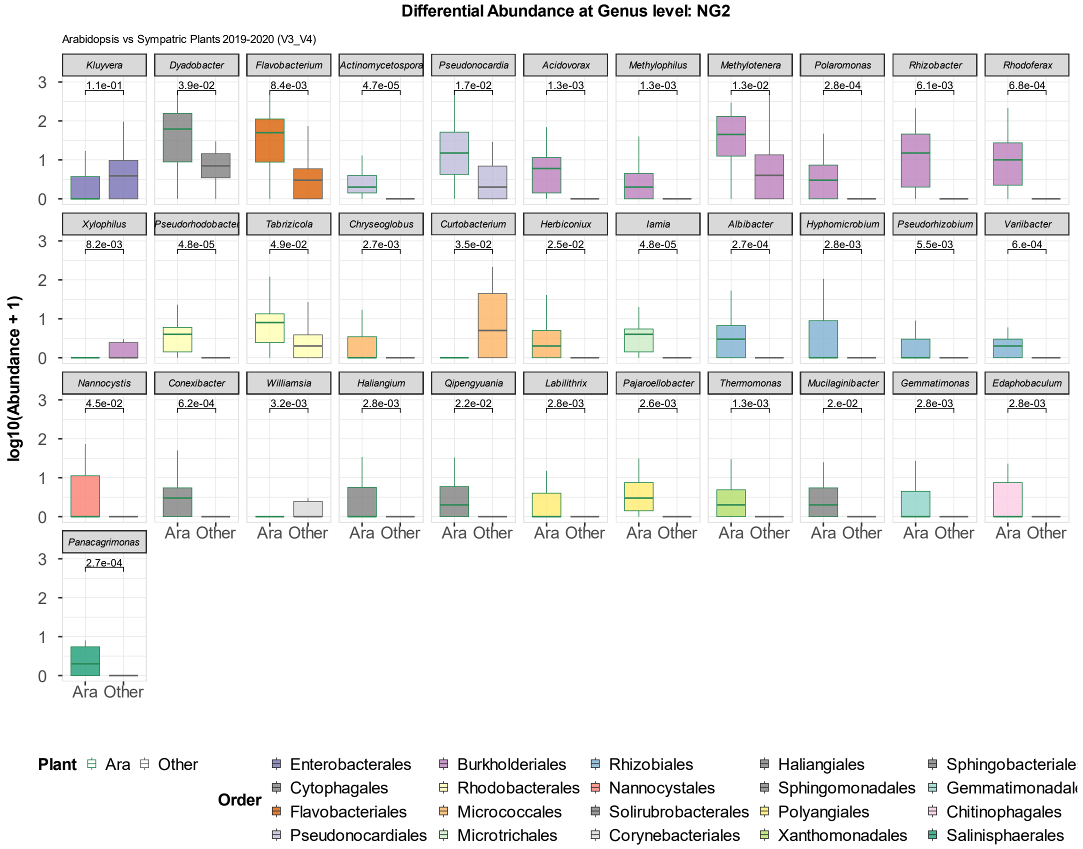
Differential Abundance at Genus level: NG2
Arabidopsis vs Sympatric Plants 2019-2020 (V3_V4)
log10(Abundance + 1)
Kluyvera 1.1e-01
Dyadobacter 3.9e-02
Flavobacterium 8.4e-03
Actinomycetospora 4.7e-05
Pseudonocardia 1.7e-02
Acidovorax 1.3e-03
Methylophilus 1.3e-03
Methylotenera 1.3e-02
Polaromonas 2.8e-04
Rhizobacter 6.1e-03
Rhodoferax 6.8e-04
Xylophilus 8.2e-03
Pseudorhodobacter 4.8e-05
Tabrizicola 4.9e-02
Chryseoglobus 2.7e-03
Curtobacterium 3.5e-02
Herbiconiux 2.5e-02
Iamia 4.8e-05
Albibacter 2.7e-04
Hyphomicrobium 2.8e-03
Pseudorhizobium 5.5e-03
Variibacter 6.e-04
Nannocystis 4.5e-02
Conexibacter 6.2e-04
Williamsia 3.2e-03
Haliangium 2.8e-03
Qipengyuania 2.2e-02
Labilithrix 2.8e-03
Pajaroellobacter 2.6e-03
Thermomonas 1.3e-03
Mucilaginibacter 2.e-02
Gemmatimonas 2.8e-03
Edaphobaculum 2.8e-03
Panacagrimonas 2.7e-04
Ara Other
Plant Ara Other
Order
Enterobacterales
Cytophagales
Flavobacteriales
Pseudonocardiales
Burkholderiales
Rhodobacterales
Micrococcales
Microtrichales
Rhizobiales
Nannocystales
Solirubrobacterales
Corynebacteriales
Haliangiales
Sphingomonadales
Polyangiales
Xanthomonadales
Sphingobacteriale
Gemmatimonadal
Chitinophagales
Salinisphaerales

B

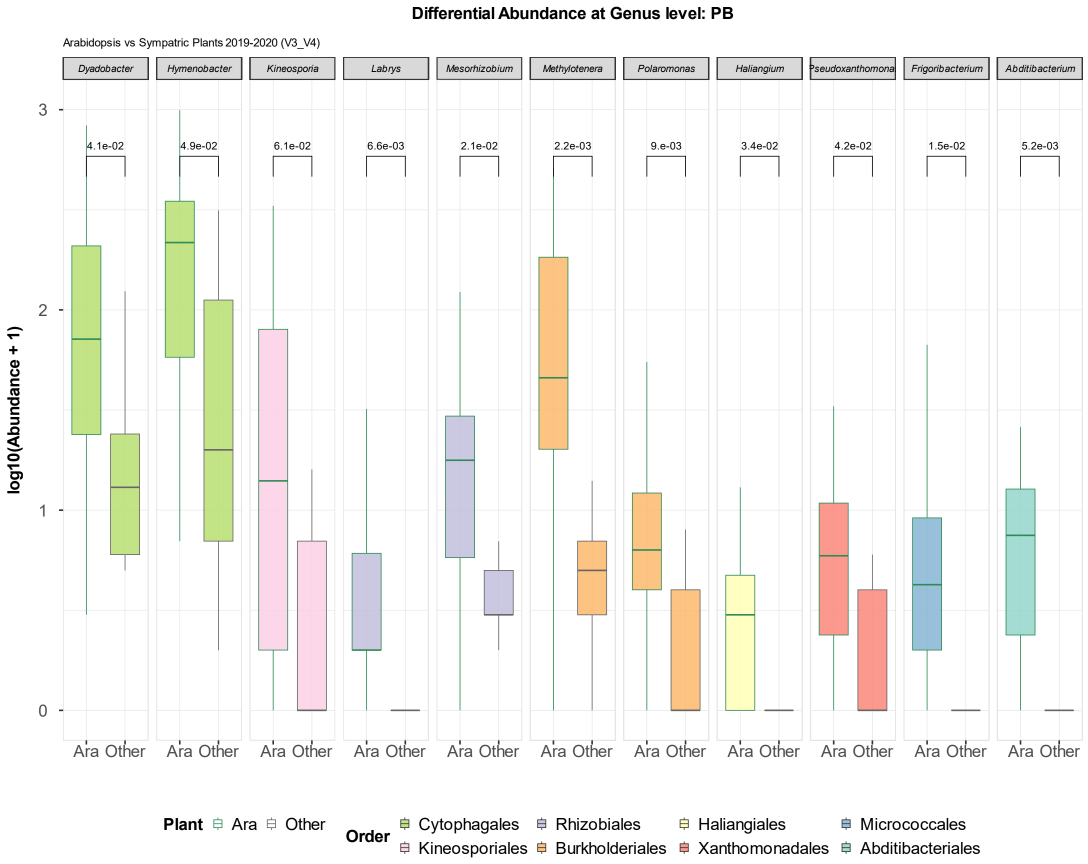

The figure is ongoing on the next page.

The figure is ongoing from the previous page.

C

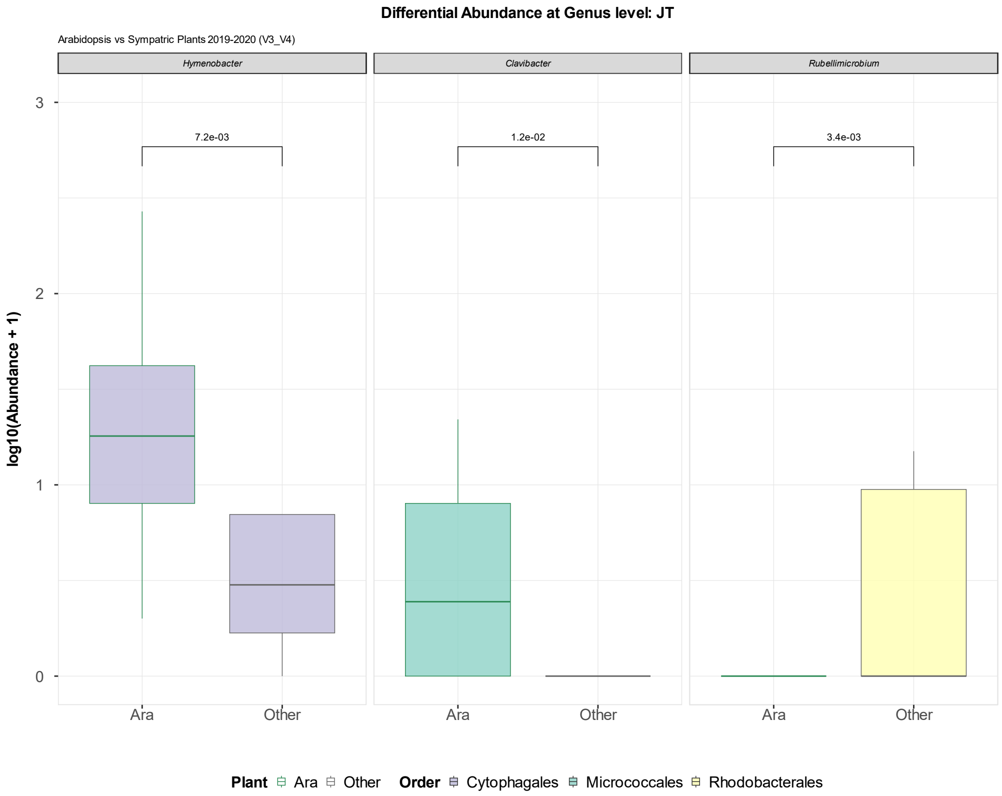

D

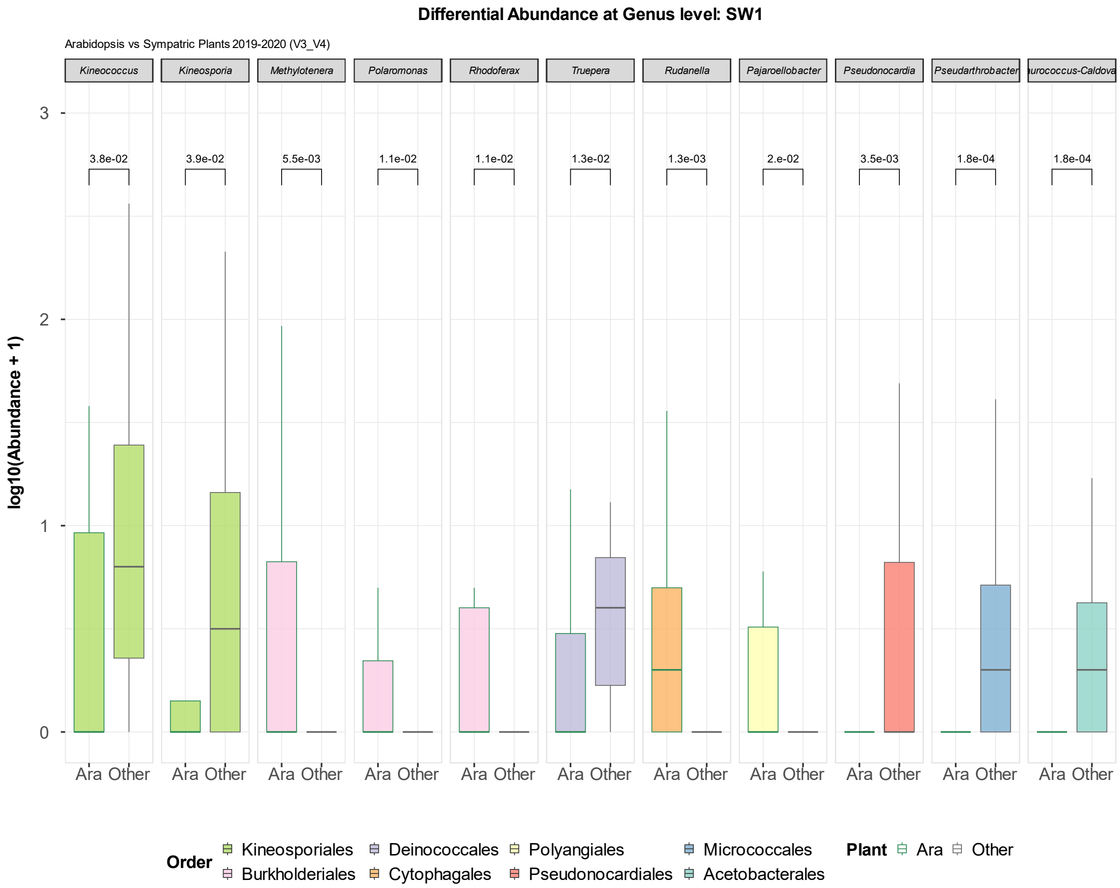

The figure is ongoing on the next page.

The figure is ongoing from the previous page.

E

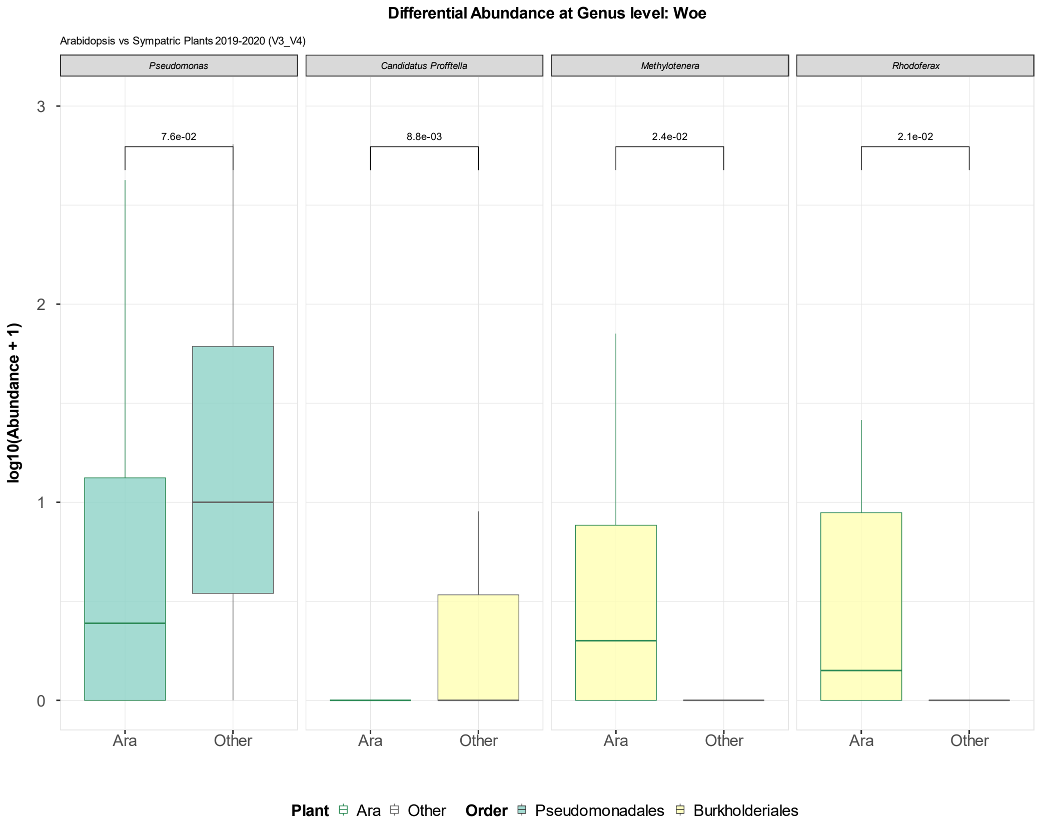

Supplementary Figure 8.

Differential abundant taxa in leaf bacterial communities from *A. thaliana* compared to sympatric non-Brassicaceae plants across the five Jena locations in February and March 2019 and 2020. Samplings with < 3 replicates were excluded and low abundance taxa were filtered out. Data is agglomerated on genus level and the pseudocount of 1 was added to each sample prior to DESeq analysis (cutoff alpha = 0.05). A Wald test with a parametric fit was executed to determine significant differences. All significantly different taxa are plotted with p-values referring to differences in abundance based on Benjamini-Hochberg adjustment. The five locations were visualized separately (**A**) NG2, (**B**) PB, (**C**) JT1, (**D**) SW1 and (**E**) Woe.

A

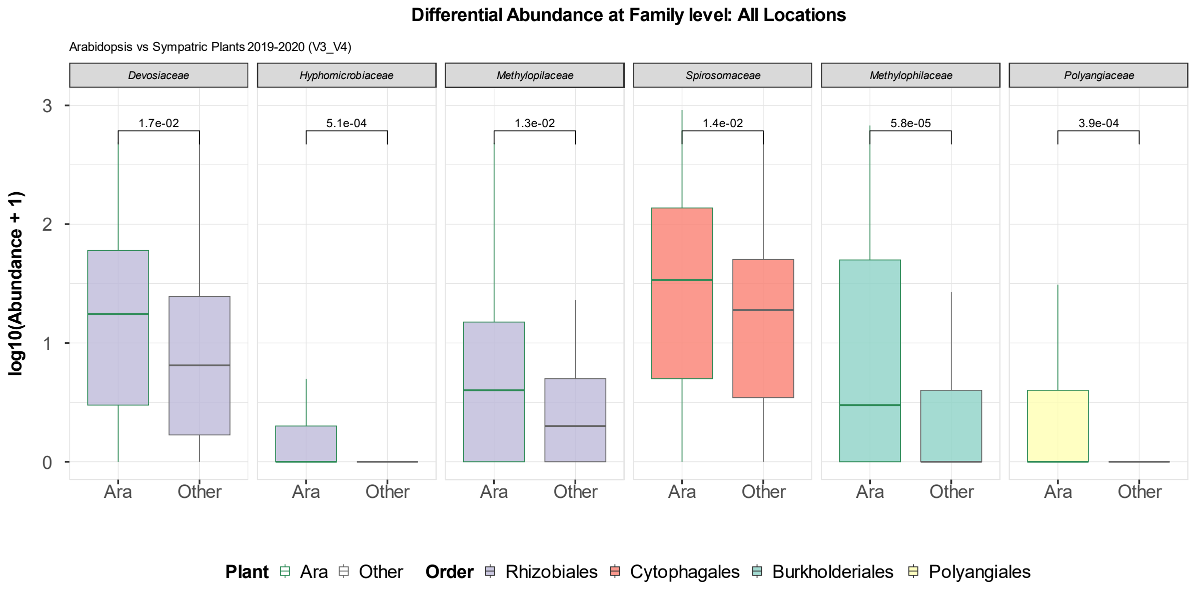

B

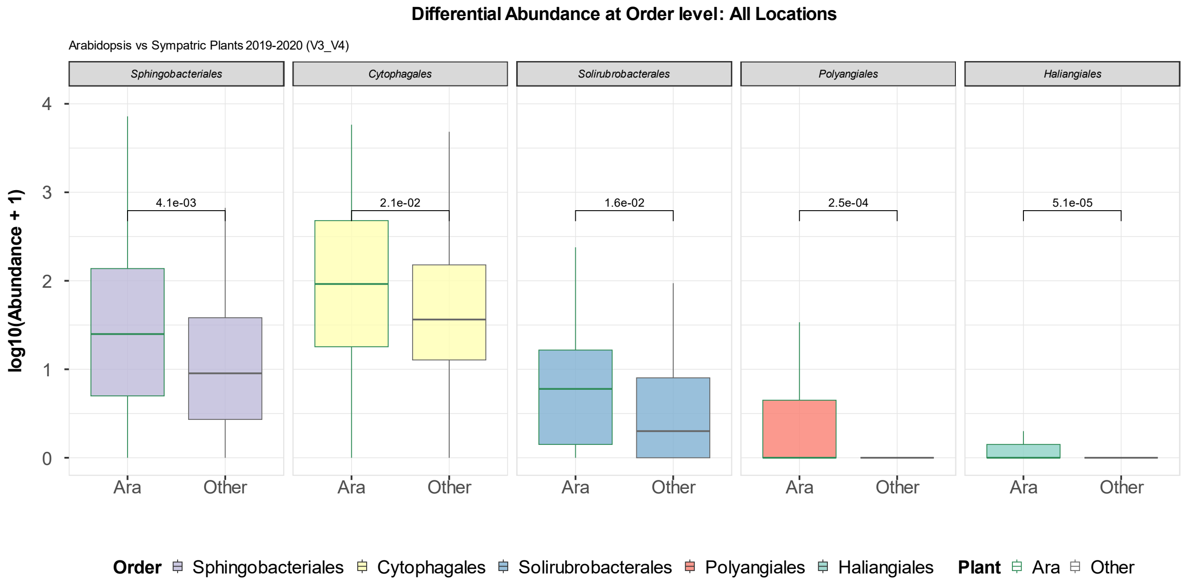

Supplementary Figure 9.

Differential abundant taxa in leaf bacterial communities from *A. thaliana* compared to sympatric non-Brassicaceae plants across the five Jena locations in February and March 2019 and 2020. Samplings with < 3 replicates were excluded and low abundance taxa were filtered out. The pseudocount of 1 was added to each sample prior to DESeq analysis (cutoff alpha = 0.05). A Wald test with a parametric fit was executed to determine significant differences. All significantly different taxa are plotted with p-values referring to differences in abundance based on Benjamini-Hochberg adjustment. (**A**) agglomerated on family level. (**B**) agglomerated on order level.

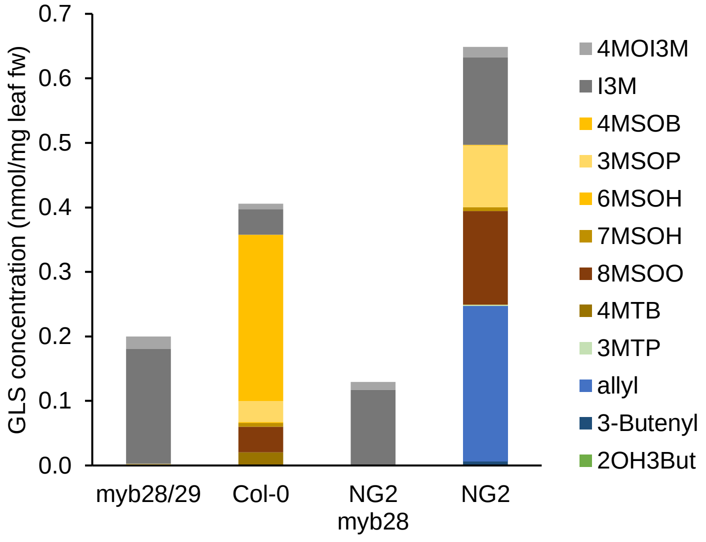

Supplementary Figure 10.

Surface GLSs of WT NG2 and Col-0 and their aliphatic GLS-free mutants. Leaves of 3-week-old plants were dipped in methanol to extract surface GLSs. Each bar shows the average of four leaves per genotype. Intact GLSs were measured on a HPLC-MS/MS. Indole GLSs are color-coded in grey. Abbreviations of GLSs are described in the Supplementary methods section. All data is available in our figshare folder (see Data Availability).

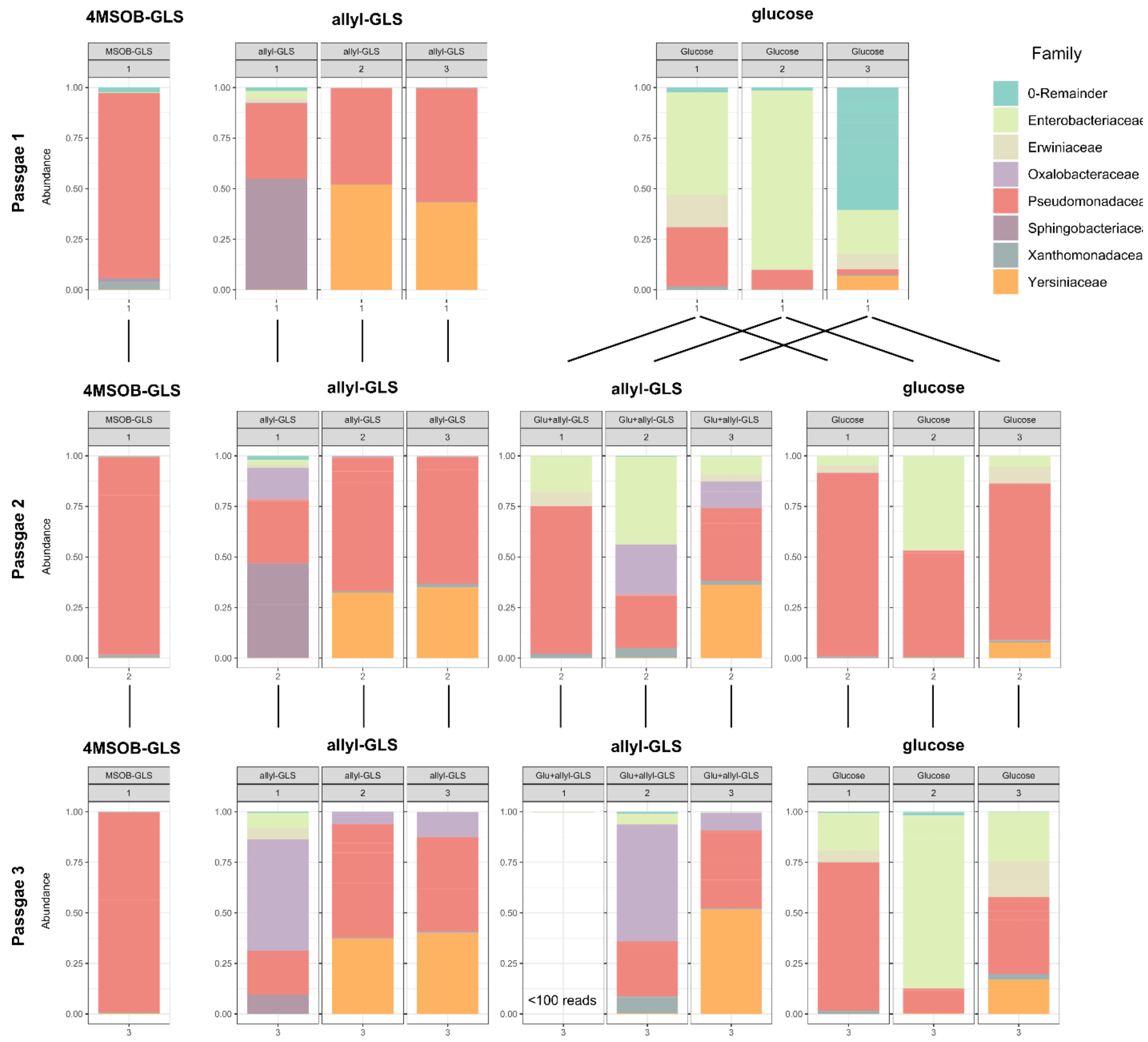

Supplementary Figure 11.

Details on the enrichment of bacterial strains from NG2 leaf surface in M9 medium supplemented with different aliphatic GLSs as sole carbon source. The bar charts show the community composition (relative abundance) after each passage assessed by 16S rRNA gene amplicon sequencing. The charts show data agglomerated on family level. Families below 0.02% relative abundance were merged and classified as "Remainder". Only replicates with >100 reads were considered. The lines between the passages show which replicate of the previous passage served as inoculum for the following passage, i.e. glucose replicates from passage 1 were used as inoculum for fresh medium with allyl-GLS as well as glucose in passage 2. All data is available in our figshare folder (see Data Availability).

A

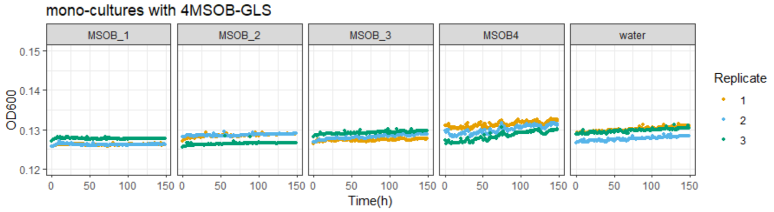

B

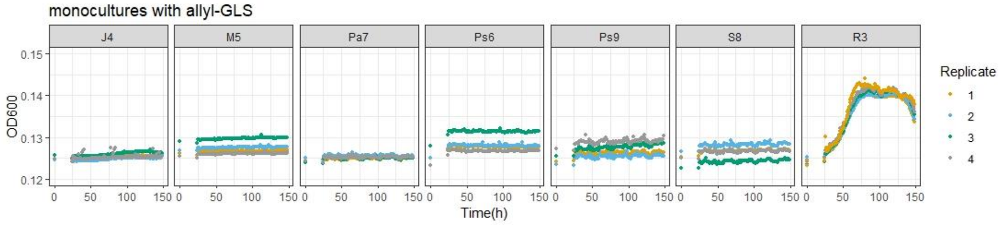

C

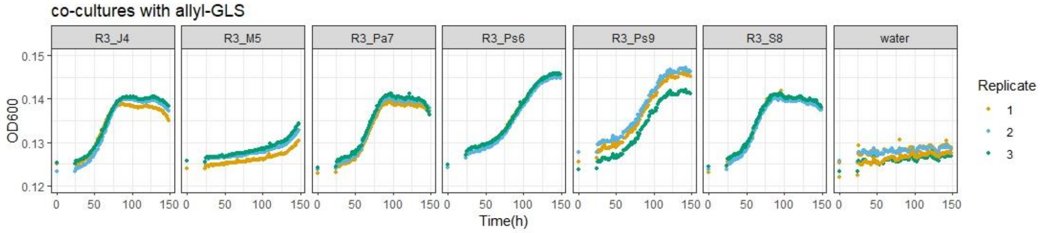

Supplementary Figure 12.

Growth of bacterial isolates on glucose and aliphatic GLSs. All isolates were grown in M9 medium supplemented with 10 mM C-source in one 6-day passage. OD_{600} was measured every hour, inoculation of the medium with water served as negative control, n=3 (A,C), n=4 (B). (**A**) Mono-cultures of *Pseudomonas* strains with 4MSOB-GLS from leaf enrichment in M9 with 4MSOB-GLS. (**B**) Mono-cultures in media supplemented with allyl-GLS of all seven isolates that were recovered from allyl-GLS enrichments, data of the first 24 h is missing. (**C**) Co-cultures of the same isolates shown in B with R3 with allyl-GLS, data of the first 24 h is missing. All data is available in our figshare folder (see Data Availability).

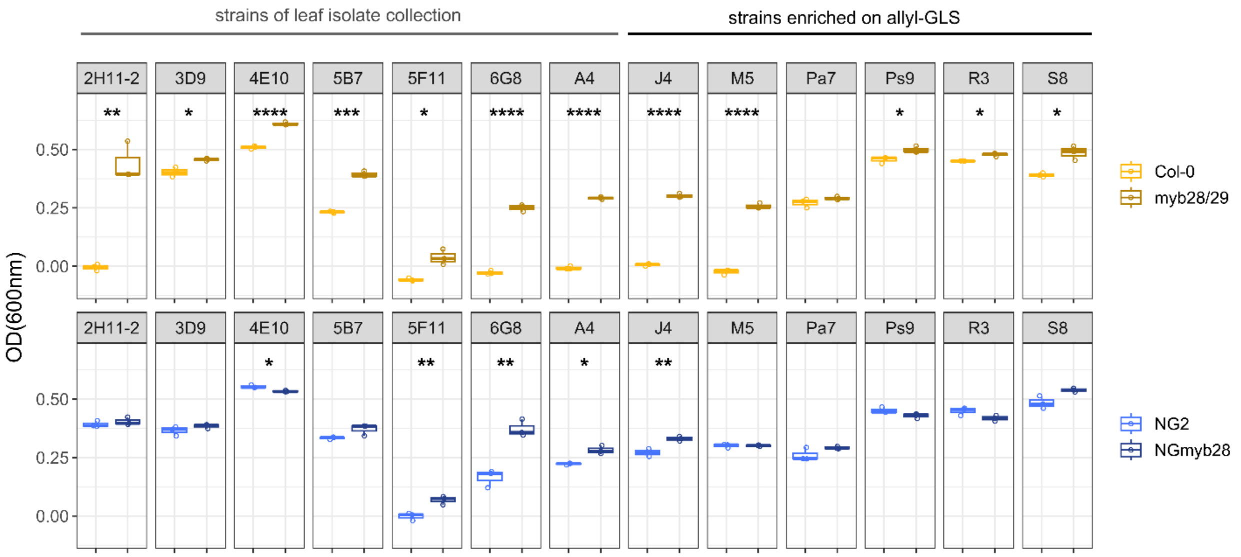

Supplementary Figure 13.

Bacterial growth in leaf extract medium. Final OD_{600} of bacterial strains grown in leaf extract medium of Col-0 and *myb28/myb29* or NG2 and NG*myb28*. The tested strains were either isolated as part of our leaf isolate collection (left) or enriched in M9 medium supplemented with allyl-GLS as sole carbon source (right). Three replicate wells were incubated at 30°C, 150 rpm shaking for 24 h. They grew in leaf media of the two *A. thaliana* genotypes NG2 and Col-0 and respective aliphatic GLS-free mutants. Each strain was grown in three technical replicates and each well was measured with multiple readings, the average per replicate is shown. Two-sided t-tests were performed and significant differences are marked with stars (*<0.05, **<0.01, ***<0.001, ****<0.0001). All data is available in our figshare folder (see Data Availability). 2H11-2 = *Plantibacter* sp., 3D9 = *Pseudomonas syringae*, 4E10 = *Xanthomonas campestris*, 5B7 = *Curtobacterium* sp., 5F11 = *Rhodococcus* sp., 6G8 = *Rhodococcus* sp., A4 = *Acidovorax* sp., J4 = *Janthinobacterium* sp., M5 = *Microbacterium* sp., Pa7 = *Pantoea* sp., Ps9 = *Pseudomonas* sp., R3 = Yersiniaceae, S8 = *Stenotrophomonas* sp.

A

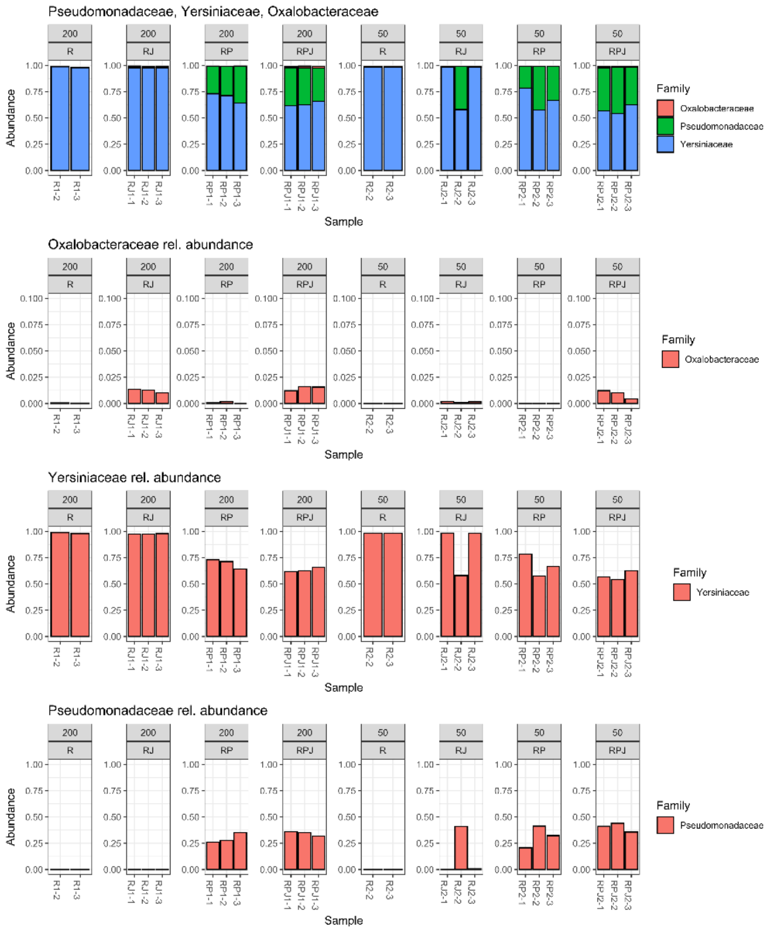

B

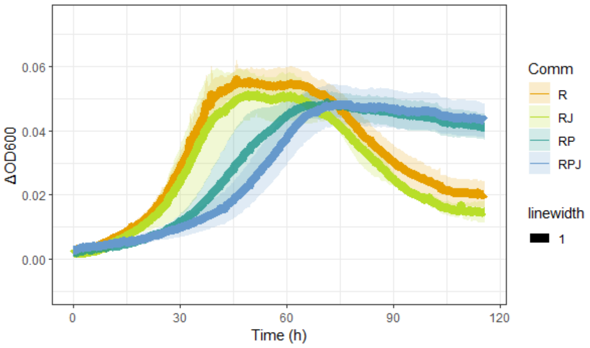

Supplementary Figure 14.

Composition of bacterial synthetic communities (SynComs) grown in three passages in M9 medium with allyl-GLS. SynComs consisted of R3, Ps9, or J4 in all possible combinations which contained R3. Three replicates were screened for each combinations, however, since R3 did not grow in one replicate, this had to be excluded from further analyses. (**A**) Relative abundances of Yersiniacaeae (R3), Pseudomonadaceae (Ps9), Oxalobacteraceae (J4), in 16S rRNA gene amplicon sequencing of final communities of two experiments: SynComs were either grown in 200 µL total volume to sample for non-targeted metabolomics (left side); or in 50 µL to report growth curves (right side). The upper panel shows stacked bar charts, the lower three relative abundances for individual families. (**B**) Average and standard deviation of the blanked OD_{600} of each SynCom in 50 µL total volume are shown. One replicate (RJ-2) was removed as it was contaminated with Ps9 according to the amplicon sequencing results (see A). All data is available in our figshare folder (see Data Availability).

Supplementary Table 1.

The five local *A. thaliana* populations identified in the city of Jena, Germany.

Name	Date found	Date collected	Name in NASC database
NG2	Fall 2017	09.05.2018	Je-1
PB	Fall 2017	13.03.2018	Not deposited yet
Woe	05.04.2018	05.04.2018	Je-3
SW1	12.04.2018	17.04.2018	Je-4
JT1	17.04.2018	17.04.2018	Je-2

Supplementary Table 2.

***sax* gene content in draft whole genomes of leaf colonizers.** Annotations are based on the genome of *Pseudomonas syringae* pv. tomato DC3000. Number of X represent the number of annotated gene copies.

ID	Isolate	*saxA*	*saxC*	*saxB*	*saxD*	*saxF*	*saxG*
5B7	*Curtobacterium* sp.						
1F1	*Curtobacterium* sp.						
2H11-2	*Plantibacter* sp.						
LR-4298	*Xanthomonas campestris*			X	XXX		
AR-41D3	*Xanthomonas campestris*			X	XXX		
4E10	*Xanthomonas campestris*				XXX	X	
LR-512C6	*Pseudomonas koreensis*			X	XX	X	X
AR-4105	*Pseudomonas syringae*	X	X	X	XXXX	X	X
3D9	*Pseudomonas syringae*	X	X	X	XX	X	X
SrG	*Stenotrophomonas sp.*						

Supplementary Table 3.

All isolates recovered from NG2 leaf wash and isolated after enrichment in M9 medium supplemented with either 4MSOB-GLS or allyl-GLS.

ID	family	genus	Top hit species	enriched on	Date of isolation	Used for an exp.?
**M1**	Microbacteriaceae	*Microbacterium*	*algeriense*	M9+allyl-GLS	17.04.2023	No
**J2**	Oxalobacteraceae	*Janthinobacterium*	*lividum*	M9+allyl-GLS	17.04.2023	No
**R3**	Yersiniaceae	*Serratia*	sp.	M9+allyl-GLS	17.04.2023	Yes
**J4**	Oxalobacteraceae	*Janthinobacterium*	*lividum*	M9+allyl-GLS	17.04.2023	Yes
**M5**	Microbacteriaceae	*Microbacterium*	*algeriense*	M9+allyl-GLS	17.04.2023	Yes
**Ps6**	Pseudomonadaceae	*Pseudomonas*	*allii*	M9+allyl-GLS	17.04.2023	Yes
**Pa7**	Erwiniaceae	*Pantoea*	*agglomerans*	M9+allyl-GLS	17.04.2023	Yes
**S8**	Xanthomondaceae	*Stenotrophomonas*	*bentonitica*	M9+allyl-GLS	17.04.2023	Yes
**Ps9**	Pseudomonadaceae	*Pseudomonas*	*lurida*	M9+allyl-GLS	17.04.2023	Yes
**MSOB1**	Pseudomonadaceae	*Pseudomonas*	*germanica*	M9+4MSOB-GLS	07.05.2023	Yes
**MSOB2**	Pseudomonadaceae	*Pseudomonas*	*germanica*	M9+4MSOB-GLS	07.05.2023	Yes
**MSOB3**	Pseudomonadaceae	*Pseudomonas*	*germanica*	M9+4MSOB-GLS	07.05.2023	Yes
**MSOB4**	Pseudomonadaceae	*Pseudomonas*	*baetica*	M9+4MSOB-GLS	07.05.2023	Yes

Supplementary Table 4.
Primers used in this study.

Name	Sequence	Target / used in this study	Source
myb28_2315F	TCTTGGTCTCCGATCTATC	*myb28* gene in NG2 / Confirmation of truncation	This study
myb28_2316R	GCTACCTTCCATGGAAGCC	*myb28* gene in NG2 / Confirmation of truncation	This study
8F	AGAGTTTGATCCTGGCTCAG	Bacterial 16S rRNA gene / Identification of 2019 isolates	(*22*)
1492R	GGTTACCTTGTTACGACTT	Bacterial 16S rRNA gene / Identification of 2019 isolates	(*22*)
799F	AACMGGATTAGATACCCKG	Bacterial 16S rRNA gene / Identification of 2018 isolates	(*23*)
1391R	GACGGGCGGTGWGTRCA	Bacterial 16S rRNA gene / Identification of 2018 isolates	(*24*)
GI_At_F1	TCCCTACACGACGCTCTTCCGATCTGTAAAGATAAATGGGTCATCTAA	*A. thaliana GI* gene / 1st PCR, amplicon sequencing	(*20*)
GI_At_R1	GGAGTTCAGACGTGTGCTCTTCCGATCTTCCTTCTGAACCGGTGTATTC	*A. thaliana GI* gene / 1st PCR, amplicon sequencing	(*20*)
341F-OH	TCCCTACACGACGCTCTTCCGATCTGACCTACGGGAGGCAGCAG	16S rRNA gene / 1st PCR, amplicon sequencing	(*20*)
799R-OH	GGAGTTCAGACGTGTGCTCTTCCGATCTTGCMGGGTATCTAATCCKGTT	16S rRNA gene / 1st PCR, amplicon sequencing	(*20*)
Indexing_Primers_fwd	AATGATACGGCGACCACCGAGATCTACACXXXXXXXXXXXXACACTCTTTCCCTACACGACGCTCTTC	16S rRNA gene / 2nd PCR, amplicon sequencing	Modified from (*20*)
Indexing_Primers_rvs	CAAGCAGAAGACGGCATACGAGATXXXXXXXXXXXXGTGACTGGAGTTCAGACGTGTGCTC	16S rRNA gene / 2nd PCR, amplicon sequencing	Modified from (*20*)
At_BLc_16S_F5	AACTTCTTTTCCAGAGAAGAAGCAAT	*A. thaliana*-specific blocking oligos / 1st PCR, amplicon sequencing	(*25*)
At_BLc_16S_R1	GCTTTCGCCGTTGGTGTTCTTTCCGATCTC	*A. thaliana*-specific blocking oligos / 1st PCR, amplicon sequencing	(*25*)

Supplementary Table 5.

Amplicon sequencing experiments performed as part of this study. n.a. = not applicable.

ID	Experiment & Figure	Leaf treat-ment	DNA extraction and purification	Plant GI gene	Primers used in 1st PCR	Blocking oligos used in 1st PCR
Data set 1	Bacterial communities in minimal medium enriched with different C-sources (Fig. 5)	n.a.	SDS buffer protocol, RNAse and proteinase K clean-up, phenol/chloroform and phenol/chloroform/isoamyl alcohol clean-up, DNA precipitation	n.a.	341F-OH 799R-OH	n.a.
Data set 2	Bacterial communities in leaves of lab-grown NG2, NG*myb28*, Col-0 and *myb28/myb29* plants (Fig. 3)	Washing, or surface-sterilizing	CTAB buffer protocol, phenol/chloroform/isoamyl alcohol clean-up, DNA precipitation	Yes	341F-OH 799R-OH	At_BLc_16S_F5 At_BLc_16S_R1
Data set 3	Bacterial communities in leaves of wild plants from the five Jena populations and co-occuring non-Brassicaceae plants, sampled in 2019 and 2020 (Fig. 4)	Washing	CTAB buffer protocol, simple DNA precipitation, magnetic bead clean-up	No	341F-OH 799R-OH	At_BLc_16S_F5 At_BLc_16S_R1
Data set 4	Bacterial synthetic communities cultured in minimal medium with allyl-GLS (Fig. 7)	n.a.	SDS buffer protocol (without SDS precipitation), phenol/chloroform and phenol/chloroform/isoamyl alcohol clean up, DNA precipitation	n.a.	341F-OH 799R-OH	n.a.

Supplementary Table 6.

Details of the analysis of GLSs by LC-MS/MS. GLSs were analyzed using an Agilent HPLC 1200/API3200 (AB SCIEX) instrument in negative ionisation mode. Abbreviations are: Q1, selected *m/z* of the first quadrupole; Q3, selected *m/z* of the third quadrupole; RT, retention time; DP, declustering potential (V); and CE, collision energy (V).

Q1	Q3	RT (min)	compound	DP	CE
436	95.8	8.6	4MSOB-GLS	-65	-60
358	95.9	10.0	Allyl-GLS	-65	-60
388	95.9	9.0	2OH3Butenyl-GLS	-65	-60
422	95.9	6.8	3MSOP-Gls	-65	-60
464	95.8	15.0	6MSOH-Gls	-65	-60
478	95.8	18.5	7MSOH-Gls	-65	-60
492	95.8	21.0	8MSOO-Gls	-75	-58
406	95.9	16.5	3MTP-Gls	-60	-58
420	95.9	19.9	4MTB-Gls	-60	-58
372	95.9	14.0	3-Butenyl	-65	-60
447	95.8	21.9	I3M-Gls	-65	-50
477	95.8	25.4	4MOI3M	-65	-50

Supplementary Table 7.

Details of the analysis of amines and 4MSOB-ITC by LC-MS/MS. 4MSOB-ITC was measured using an Agilent HPLC 1200/API3200 (AB SCIEX) instrument in positive ionisation mode. Abbreviations are: Q1, selected *m/z* of the first quadrupole; Q3, selected *m/z* of the third quadrupole; RT, retention time; DP, declustering potential (V); and CE, collision energy (V); prop-2-en-1-amine is the amine formed from allyl-GLS; 1-aminobut-3-en-2-ol is the amine formed from 2-OH-3-Butenyl-GLS.

Q1	Q3	RT (min)	compound	DP	CE
136	72	0.5	4MSOB-amine	26	17
58	41	0.5	prop-2-en-1-amine	21	13
88	71	0.6	1-aminobut-3-en-2-ol	21	13
178	114	2.6	4MSOB-ITC	60	13

Supplementary References

1. N. Rohland, D. Reich, Cost-effective, high-throughput DNA sequencing libraries for multiplexed target capture. *Genome Res.* **22**, 939–946 (2012).

2. A. M. Bolger, M. Lohse, B. Usadel, Trimmomatic: a flexible trimmer for Illumina sequence data. *Bioinformatics* **30**, 2114–2120 (2014).

3. S. Nurk, A. Bankevich, D. Antipov, A. Gurevich, A. Korobeynikov, A. Lapidus, A. Prjibelsky, A. Pyshkin, A. Sirotkin, Y. Sirotkin, R. Stepanauskas, J. McLean, R. Lasken, S. R. Clingenpeel, T. Woyke, G. Tesler, M. A. Alekseyev, P. A. Pevzner, "Assembling Genomes and Mini-metagenomes from Highly Chimeric Reads" in *Research in Computational Molecular Biology*, M. Deng, R. Jiang, F. Sun, X. Zhang, Eds. (Springer, Berlin, Heidelberg, 2013)*Lecture Notes in Computer Science*, pp. 158–170.

4. T. Seemann, Prokka: rapid prokaryotic genome annotation. *Bioinformatics* **30**, 2068–2069 (2014).

5. T. J. M. van den Bosch, K. Tan, A. Joachimiak, C. U. Welte, Functional Profiling and Crystal Structures of Isothiocyanate Hydrolases Found in Gut-Associated and Plant-Pathogenic Bacteria. *Appl. Environ. Microbiol.* **84**, e00478-18 (2018).

6. F. Teufel, J. J. Almagro Armenteros, A. R. Johansen, M. H. Gíslason, S. I. Pihl, K. D. Tsirigos, O. Winther, S. Brunak, G. von Heijne, H. Nielsen, SignalP 6.0 predicts all five types of signal peptides using protein language models. *Nat. Biotechnol.* **40**, 1023–1025 (2022).

7. D. J. Kliebenstein, J. Kroymann, P. Brown, A. Figuth, D. Pedersen, J. Gershenzon, T. Mitchell-Olds, Genetic control of natural variation in Arabidopsis glucosinolate accumulation. *Plant Physiol.* **126**, 811–825 (2001).

8. M. Burow, R. Müller, J. Gershenzon, U. Wittstock, Altered glucosinolate hydrolysis in genetically engineered Arabidopsis thaliana and its influence on the larval development of Spodoptera littoralis. *J. Chem. Ecol.* **32**, 2333–2349 (2006).

9. P. D. Brown, J. G. Tokuhisa, M. Reichelt, J. Gershenzon, Variation of glucosinolate accumulation among different organs and developmental stages of Arabidopsis thaliana. *Phytochemistry* **62**, 471–481 (2003).

10. M. de Vos, K. L. Kriksunov, G. Jander, Indole-3-acetonitrile production from indole glucosinolates deters oviposition by Pieris rapae. *Plant Physiol.* **146**, 916–926 (2008).

11. Z.-L. Yang, F. Seitz, V. Grabe, S. Nietzsche, A. Richter, M. Reichelt, R. Beutel, F. Beran, Rapid and Selective Absorption of Plant Defense Compounds From the Gut of a Sequestering Insect. *Front. Physiol.* **13**, 846732 (2022).

12. U. Wittstock, K. Meier, F. Dörr, B. M. Ravindran, NSP-Dependent Simple Nitrile Formation Dominates upon Breakdown of Major Aliphatic Glucosinolates in Roots, Seeds, and Seedlings of Arabidopsis thaliana Columbia-0. *Front. Plant Sci.* **7**, 1821 (2016).

13. V. Lambrix, M. Reichelt, T. Mitchell-Olds, D. J. Kliebenstein, J. Gershenzon, The Arabidopsis epithiospecifier protein promotes the hydrolysis of glucosinolates to nitriles and influences Trichoplusia ni herbivory. *Plant Cell* **13**, 2793–2807 (2001).

14. J. T. Scanlon, D. E. Willis, Calculation of Flame Ionization Detector Relative Response Factors Using the Effective Carbon Number Concept. *J. Chromatogr. Sci.* **23**, 333–340 (1985).

15. P. Medina-van Berkum, E. Schmöckel, A. Bischoff, N. Carrasco-Farias, J. A. Catford, R. Feldmann, K. Groten, H. A. L. Henry, A. Bucharova, S. Hänniger, J. C. Luong, J. Meis, V. S. P. Oetama, M. Pärtel, S. A. Power, J. Villellas, E. Welk, A. Wingler, B. Rothe, J. Gershenzon, M. Reichelt, C. Roscher, S. B. Unsicker, Plant geographic distribution influences chemical defences in native and introduced Plantago lanceolata populations. *Funct. Ecol.* **38**, 883–896 (2024).

16. L.-F. Nothias, D. Petras, R. Schmid, K. Dührkop, J. Rainer, A. Sarvepalli, I. Protsyuk, M. Ernst, H. Tsugawa, M. Fleischauer, F. Aicheler, A. A. Aksenov, O. Alka, P.-M. Allard, A. Barsch, X. Cachet, A. M. Caraballo-Rodriguez, R. R. Da Silva, T. Dang, N. Garg, J. M. Gauglitz, A. Gurevich, G. Isaac, A. K. Jarmusch, Z. Kameník, K. B. Kang, N. Kessler, I. Koester, A. Korf, A. Le Gouellec, M. Ludwig, C. Martin H, L.-I. McCall, J. McSayles, S. W. Meyer, H. Mohimani, M. Morsy, O. Moyne, S. Neumann, H. Neuweger, N. H. Nguyen, M. Nothias-Esposito, J. Paolini, V. V. Phelan, T. Pluskal, R. A. Quinn, S. Rogers, B. Shrestha, A. Tripathi, J. J. J. van der Hooft, F. Vargas, K. C. Weldon, M. Witting, H. Yang, Z. Zhang, F. Zubeil, O. Kohlbacher, S. Böcker, T. Alexandrov, N. Bandeira, M. Wang, P. C. Dorrestein, Feature-based molecular networking in the GNPS analysis environment. *Nat. Methods* **17**, 905–908 (2020).

17. K. Dührkop, M. Fleischauer, M. Ludwig, A. A. Aksenov, A. V. Melnik, M. Meusel, P. C. Dorrestein, J. Rousu, S. Böcker, SIRIUS 4: a rapid tool for turning tandem mass spectra into metabolite structure information. *Nat. Methods* **16**, 299–302 (2019).

18. M. Ludwig, L.-F. Nothias, K. Dührkop, I. Koester, M. Fleischauer, M. A. Hoffmann, D. Petras, F. Vargas, M. Morsy, L. Aluwihare, P. C. Dorrestein, S. Böcker, Database-independent molecular formula annotation using Gibbs sampling through ZODIAC. *Nat. Mach. Intell.* **2**, 629–641 (2020).

19. K. Dührkop, L.-F. Nothias, M. Fleischauer, R. Reher, M. Ludwig, M. A. Hoffmann, D. Petras, W. H. Gerwick, J. Rousu, P. C. Dorrestein, S. Böcker, Systematic classification of unknown metabolites using high-resolution fragmentation mass spectra. *Nat. Biotechnol.* **39**, 462–471 (2021).

20. D. S. Lundberg, P. Pramoj Na Ayutthaya, A. Strauß, G. Shirsekar, W.-S. Lo, T. Lahaye, D. Weigel, Host-associated microbe PCR (hamPCR) enables convenient measurement of both microbial load and community composition. *eLife* **10**, e66186 (2021).

21. M. I. Love, W. Huber, S. Anders, Moderated estimation of fold change and dispersion for RNA-seq data with DESeq2. *Genome Biol.* **15**, 550 (2014).

22. S. Turner, K. M. Pryer, V. P. Miao, J. D. Palmer, Investigating deep phylogenetic relationships among cyanobacteria and plastids by small subunit rRNA sequence analysis. *J. Eukaryot. Microbiol.* **46**, 327–338 (1999).

23. M. K. Chelius, E. W. Triplett, The Diversity of Archaea and Bacteria in Association with the Roots of Zea mays L. *Microb. Ecol.* **41**, 252–263 (2001).

24. J. J. Walker, N. R. Pace, Phylogenetic composition of Rocky Mountain endolithic microbial ecosystems. *Appl. Environ. Microbiol.* **73**, 3497–3504 (2007).

25. T. Mayer, A. Mari, J. Almario, M. Murillo-Roos, H. Syed M Abdullah, N. Dombrowski, S. Hacquard, E. M. Kemen, M. T. Agler, Obtaining deeper insights into microbiome diversity using a simple method to block host and nontargets in amplicon sequencing. *Mol. Ecol. Resour.*, doi: 10.1111/1755-0998.13408 (2021).
