# Supplementary material for: Glucosinolate structural diversity shapes recruitment of a metabolic network of leaf-associated bacteria
Source: Nat Commun. 2024 Oct 1;15:8496. doi: 10.1038/s41467-024-52679-7 (PMC11445407; doi:10.1038/s41467-024-52679-7)
Supplement: Supplementary file 3 — Description of Additional Supplementary Files [file 41467_2024_52679_MOESM3_ESM.pdf]

<u>Description of Additional Supplementary Files</u>

Supplementary Data 1. All strains isolated from A. thaliana leaves and used in this study.

Supplementary Data 2. Statistics for alpha diversity and beta diversity of bacterial communities in lab-grown NG2 –NGmyb28, Col-0 – myb28/myb29 plants (see Fig. 3).

Supplementary Data 3. Results of BLAST search for putative myrosinase and SaxA homologs in the genome of R3

Supplementary Data 4. Number of samples and results of Tukey's Post-hoc test performed for leaf extract growth experiment (see Fig. 2A).

Supplementary Data 5. Peak statistics of pairwise comparisons of R3 mono-culture with co-cultures including R3 (see Fig. 7F).

Supplementary Data 6. Results of Tukey's Post-hoc test performed for allyl-ITC and allyl-amine concentrations in cocultures (see Fig. 7G).
