# Supplementary material for: Glucosinolate structural diversity shapes recruitment of a metabolic network of leaf-associated bacteria
Source: Nat Commun. 2024 Oct 1;15:8496. doi: 10.1038/s41467-024-52679-7 (PMC11445407; doi:10.1038/s41467-024-52679-7)
Supplement: Supplementary file 10 — Reporting Summary [file 41467_2024_52679_MOESM10_ESM.pdf]

nature portfolio

Corresponding author(s): Dr. Matthew Agler

Last updated by author(s): Jun 26, 2024

Reporting Summary

Nature Portfolio wishes to improve the reproducibility of the work that we publish. This form provides structure for consistency and transparency in reporting. For further information on Nature Portfolio policies, see our Editorial Policies and the Editorial Policy Checklist.

Statistics

For all statistical analyses, confirm that the following items are present in the figure legend, table legend, main text, or Methods section.

n/a	Confirmed	
☐	☒	The exact sample size (*n*) for each experimental group/condition, given as a discrete number and unit of measurement
☐	☒	A statement on whether measurements were taken from distinct samples or whether the same sample was measured repeatedly
☐	☒	The statistical test(s) used AND whether they are one- or two-sided<br>*Only common tests should be described solely by name; describe more complex techniques in the Methods section.*
☒	☐	A description of all covariates tested
☐	☒	A description of any assumptions or corrections, such as tests of normality and adjustment for multiple comparisons
☐	☒	A full description of the statistical parameters including central tendency (e.g. means) or other basic estimates (e.g. regression coefficient) AND variation (e.g. standard deviation) or associated estimates of uncertainty (e.g. confidence intervals)
☐	☒	For null hypothesis testing, the test statistic (e.g. *F*, *t*, *r*) with confidence intervals, effect sizes, degrees of freedom and *P* value noted<br>*Give P values as exact values whenever suitable.*
☒	☐	For Bayesian analysis, information on the choice of priors and Markov chain Monte Carlo settings
☒	☐	For hierarchical and complex designs, identification of the appropriate level for tests and full reporting of outcomes
☒	☐	Estimates of effect sizes (e.g. Cohen's *d*, Pearson's *r*), indicating how they were calculated

Our web collection on statistics for biologists contains articles on many of the points above.

Software and code

Policy information about availability of computer code

Data collection	*Provide a description of all commercial, open source and custom code used to collect the data in this study, specifying the version used OR state that no software was used.*
Data analysis	*Provide a description of all commercial, open source and custom code used to analyse the data in this study, specifying the version used OR state that no software was used.*

For manuscripts utilizing custom algorithms or software that are central to the research but not yet described in published literature, software must be made available to editors and reviewers. We strongly encourage code deposition in a community repository (e.g. GitHub). See the Nature Portfolio guidelines for submitting code & software for further information.

Data

Policy information about availability of data

All manuscripts must include a data availability statement. This statement should provide the following information, where applicable:

- Accession codes, unique identifiers, or web links for publicly available datasets
- A description of any restrictions on data availability
- For clinical datasets or third party data, please ensure that the statement adheres to our policy

The amplicon sequencing data generated in this study have been deposited in the NCBI-SRA database under accession codes: PRJNA1032255 [https://www.ncbi.nlm.nih.gov/bioproject/PRJNA1032255/]

PRJNA815825 [https://www.ncbi.nlm.nih.gov/bioproject/PRJNA815825]
PRJNA1124263 [https://www.ncbi.nlm.nih.gov/bioproject/PRJNA1124263].

The bacterial genome sequencing data generated in this study have been deposited in the NCBI-SRA database under accession codes:
PRJNA1124271 [https://www.ncbi.nlm.nih.gov/bioproject/PRJNA1124271].
PRJNA1152919 [https://www.ncbi.nlm.nih.gov/bioproject/PRJNA1152919].

All processed data with code to generate the main and supplementary figures are available at Figshare in our folder: [https://figshare.com/projects/Glucosinolate_structural_diversity_shapes_recruitment_of_a_metabolic_network_of_leaf-associated_bacteria/180211].
Individual items are saved at figshare with their respective DOI numbers:
[https://doi.org/10.6084/m9.figshare.26085514.v2] Data & Script for Fig. 7C.
[https://doi.org/10.6084/m9.figshare.26085520.v2] Data & Script for Fig. 7G.
[https://doi.org/10.6084/m9.figshare.26086114.v2] Data & Script for Fig. 6C, 6D.
[https://doi.org/10.6084/m9.figshare.24297421.v5] Data & Scripts for Fig. 4 and Fig. S8, S9.
[https://doi.org/10.6084/m9.figshare.26086120.v1] Data & Script for Fig. 7E, 7F.
[https://doi.org/10.6084/m9.figshare.26086123.v2] Data & Script for Fig. 6A, 7A and S12.
[https://doi.org/10.6084/m9.figshare.24242887.v5] Data & Scripts for Fig. 7D and Fig. S13A.
[https://doi.org/10.6084/m9.figshare.26022871.v2] Data & Script for Fig. S14.
[https://doi.org/10.6084/m9.figshare.26022958.v1] Data for Fig. S10.
[https://doi.org/10.6084/m9.figshare.24242881.v1] Data & Scripts for Fig. 5 and S11.
[https://doi.org/10.6084/m9.figshare.24297424.v3] Data & Scripts for Fig. 3 and Fig. S5, S6, S7.
[https://doi.org/10.6084/m9.figshare.26021752.v1] Data S3 - Homology search in R3.
[https://doi.org/10.6084/m9.figshare.24297385.v1] Data for Fig. 2D.
[https://doi.org/10.6084/m9.figshare.24297409.v2] Data & Scripts for Fig. 6B.
[https://doi.org/10.6084/m9.figshare.24242812.v1] Data for Fig. 1B.
[https://doi.org/10.6084/m9.figshare.24242854.v1] Data & Scripts for Fig. 2A and Fig. S2.
[https://doi.org/10.6084/m9.figshare.24297379.v1] Data & Scripts for Fig. 2B,C.
[https://doi.org/10.6084/m9.figshare.24297427.v1] Data for Fig. S1.
[https://doi.org/10.6084/m9.figshare.24297502.v1] Data for Fig. S3.
[https://doi.org/10.6084/m9.figshare.24523660.v1] Data for Tab S3.

Research involving human participants, their data, or biological material

Policy information about studies with human participants or human data. See also policy information about sex, gender (identity/presentation), and sexual orientation and race, ethnicity and racism.

Reporting on sex and gender	*Use the terms sex (biological attribute) and gender (shaped by social and cultural circumstances) carefully in order to avoid confusing both terms. Indicate if findings apply to only one sex or gender; describe whether sex and gender were considered in study design; whether sex and/or gender was determined based on self-reporting or assigned and methods used. Provide in the source data disaggregated sex and gender data, where this information has been collected, and if consent has been obtained for sharing of individual-level data; provide overall numbers in this Reporting Summary. Please state if this information has not been collected. Report sex- and gender-based analyses where performed, justify reasons for lack of sex- and gender-based analysis.*
Reporting on race, ethnicity, or other socially relevant groupings	*Please specify the socially constructed or socially relevant categorization variable(s) used in your manuscript and explain why they were used. Please note that such variables should not be used as proxies for other socially constructed/relevant variables (for example, race or ethnicity should not be used as a proxy for socioeconomic status). Provide clear definitions of the relevant terms used, how they were provided (by the participants/respondents, the researchers, or third parties), and the method(s) used to classify people into the different categories (e.g. self-report, census or administrative data, social media data, etc.) Please provide details about how you controlled for confounding variables in your analyses.*
Population characteristics	*Describe the covariate-relevant population characteristics of the human research participants (e.g. age, genotypic information, past and current diagnosis and treatment categories). If you filled out the behavioural & social sciences study design questions and have nothing to add here, write "See above."*
Recruitment	*Describe how participants were recruited. Outline any potential self-selection bias or other biases that may be present and how these are likely to impact results.*
Ethics oversight	*Identify the organization(s) that approved the study protocol.*

Note that full information on the approval of the study protocol must also be provided in the manuscript.

Field-specific reporting

Please select the one below that is the best fit for your research. If you are not sure, read the appropriate sections before making your selection.

☒ Life sciences ☐ Behavioural & social sciences ☐ Ecological, evolutionary & environmental sciences

For a reference copy of the document with all sections, see nature.com/documents/nr-reporting-summary-flat.pdf

Life sciences study design

All studies must disclose on these points even when the disclosure is negative.

Sample size	Sample sizes for laboratory experiments were chosen based on reproducibility of the results and feasibility. Sample sizes for the sampling from wild populations were adjusted based on availability of plants in individual populations and so may not be exactly the same in each population.
Data exclusions	16S amplicon sequencing data of leaf samples always contains plant chloroplast and mitochondria 16S reads. Depending on the bacterial load, this can make up a substantial portion of the sample data (especially in "endophyte" samples which are very low bacterial load). After removing chloroplast and mitochondria reads, we excluded samples that contained less than 100 bacterial reads, because the negative controls had always <50 reads.
Replication	The correlation of enrichment of specific taxa to glucosinolates in-planta is based on independent replicate plants. Many of the same taxa were enriched in wild A. thaliana compared to other plants over two years. Since this wild plant data is completely independent from lab experiments and subject to variation from diverse environmental factors, this represents strong orthogonal replication of the enrichment in A. thaliana. The in-vitro enrichment from wild plants only inlcludes one fully independent plant sample from the wild population, but the enrichment itself includes multiple replicates. Additionally, the following in-vitro culturing experiments with isolates from the enrichment help to confirm and explain the enrichment results, so also represent a degree of orthogonal testing of results.
Randomization	Sampling of plants was always performed randomly. Since plant "treatments" were mutations, there was no assigning of individuals to treatment groups that could have been randomized.
Blinding	Because of regulations of GMOs, plant "treatments" (mutants) need to be labeled and blinding is not possible. However, all plants were grown together in single cabinets and were treated identically.

Reporting for specific materials, systems and methods

We require information from authors about some types of materials, experimental systems and methods used in many studies. Here, indicate whether each material, system or method listed is relevant to your study. If you are not sure if a list item applies to your research, read the appropriate section before selecting a response.

Materials & experimental systems

n/a	Involved in the study	
☒	☐	Antibodies
☒	☐	Eukaryotic cell lines
☒	☐	Palaeontology and archaeology
☒	☐	Animals and other organisms
☒	☐	Clinical data
☒	☐	Dual use research of concern
☐	☒	Plants

Methods

n/a	Involved in the study	
☒	☐	ChIP-seq
☒	☐	Flow cytometry
☒	☐	MRI-based neuroimaging

Plants

Seed stocks

Col-0 seeds were from a common laboratory stock but are available via from NASC (N1092). The myb28 mutant was acquired from NASC (SALK_136312C). myb28myb29 double mutants were provided by Daniel Vassao, MPI-CE Jena, Germany. Local Jena A. thaliana genotypes NG2, JT1, Woe and SW are already deposited in NASC database (N2110865- N2110868) and PB and NGmyb28 mutant will fTo study effects of aliphatic GLSs in NG2 we generated an aliphatic GLS-free mutant in NG2 background by knocking out the Myb28 transcription factor using a genome editing procedure by an RNA-guided SpCas9 nuclease. The plasmid pDGE347 was programmed for six target sites within MYB28 (AT5G61420; AAAAAACGTTTGATGGAACAGGG; TTCAAATTCTCATCGACCGTAGG; GATCGGGAGTATTGCTTGTCGG; GCTTCTAGTTCCAACCCTACGG; GAAACCATGTTGCAACTGGATGG; GAAACGTTTCTTGCAACTCAAGG). The respective plasmid (pDGE816) was transformed into Agrobacterium tumefaciens strain GV3101 pMP90 and plants of accession NG2 were transformed by floral dipping as previously described. Floral dipping resulted in CRISPR-guided transformation events already in the germ cells of the plant and therefore T1 generation seeds were screened for successfully transformed seeds (indicated by RFP expression in seeds). Primary transformants and non-transgenic individuals from the T2 population were PCR screened and Sanger sequenced to isolate homozygous myb28 lines using oligonucleotides myb28_2315F and myb28_2316R (Tab. S4). Leaves of plants of T3 or T4 generation were used for GLS analysis to confirm the decrease in aliphatic GLS levelsollow.From 2019 to 2020, we collected A. thaliana leaf samples from the five different locations in Jena. At the same time, a similar number of other random plants were sampled. Sampling was conducted during the early days of spring in February and March each year. For smaller A. thaliana plants, we sampled half of the rosette, while for larger ones, 2-3 leaves were collected. For other plants a similar amount of plant material was selected. The leaf material was washed with sterile MiliQ water three times and samples were brought back to the lab on ice. Plant material was frozen in screw cap tubes with two metal beads and ~0.2 g glass beads (0.25-0.5 mm diameter) each at -80°C until further processing.

Novel plant genotypes

Describe the methods by which all novel plant genotypes were produced. This includes those generated by transgenic approaches, gene editing, chemical/radiation-based mutagenesis and hybridization. For transgenic lines, describe the transformation method, the number of independent lines analyzed and the generation upon which experiments were performed. For gene-edited lines, describe the editor used, the endogenous sequence targeted for editing, the targeting guide RNA sequence (if applicable) and how the editor was applied.

Authentication

We confirmed mutant stocks and CRISPR mutants using both PCR and analysis of glucosinolates. Known secondary effects of the myb28/29 mutation are dependent on ammonium stress (Coleto et al, New Phytologist, 2020), which we do not expect in our experimental setup in soil and we did not observe any other non-target effects. We also did not observe any unexpected phenotypic effects in the NG2 myb28 CRISPR mutants. Additionally, the results of the wild and in-vitro enrichment experiments corroborate the effects observed in the myb mutant plants, strongly suggesting that the observed effects were due to glucosinolates and not due to other secondary effects.
